# Nuclear receptors of the honey bee: annotation and expression in the adult brain

**DOI:** 10.1111/j.1365-2583.2006.00679.x

**Published:** 2006-10-01

**Authors:** Rodrigo A Velarde, Gene E Robinson, Susan E Fahrbach

**Affiliations:** * Department of Entomology, University of Illinois at Urbana-Champaign Urbana, Illinois, USA; † Neuroscience Program, University of Illinois at Urbana-Champaign Urbana, Illinois, USA; ‡ Department of Biology, Wake Forest University Winston-Salem, NC, USA

**Keywords:** *Apis mellifera*, photoreceptor cell specific nuclear receptor, seven-up, steroid hormone receptor, ultraspiracle.

## Abstract

The Drosophila genome encodes 18 canonical nuclear receptors. All of the Drosophila nuclear receptors are here shown to be present in the genome of the honey bee (*Apis mellifera*). Given that the time since divergence of the Drosophila and Apis lineages is measured in hundreds of millions of years, the identification of matched orthologous nuclear receptors in the two genomes reveals the fundamental set of nuclear receptors required to ‘make’ an endopterygote insect. The single novelty is the presence in the *A. mellifera* genome of a third insect gene similar to vertebrate photoreceptor-specific nuclear receptor (PNR). Phylogenetic analysis indicates that this novel gene, which we have named *AmPNR*-like, is a new member of the *NR2* subfamily not found in the Drosophila or human genomes. This gene is expressed in the developing compound eye of the honey bee. Like their vertebrate counterparts, arthropod nuclear receptors play key roles in embryonic and postembryonic development. Studies in Drosophila have focused primarily on the role of these transcription factors in embryogenesis and metamorphosis. Examination of an expressed sequence tag library developed from the adult bee brain and analysis of transcript expression in brain using *in situ* hybridization and quantitative RT-PCR revealed that several members of the nuclear receptor family (*AmSVP*, *AmUSP*, *AmERR*, *AmHr46*, *AmFtz-F1*, and *AmHnf-4*) are expressed in the brain of the adult bee. Further analysis of the expression of *AmUSP* and *AmSVP* in the mushroom bodies, the major insect brain centre for learning and memory, revealed changes in transcript abundance and, in the case of *AmUSP*, changes in transcript localization, during the development of foraging behaviour in the adult. Study of the honey bee therefore provides a model for understanding nuclear receptor function in the adult brain.

## Introduction

Nuclear receptors constitute a protein superfamily that can be recognized in metazoans as distantly related as jellyfish and humans by the presence of highly conserved N-terminal DNA-binding domains (DBD) with 2 C4 zinc fingers (Robinson-Rechavi & Laudet, 2003). Nuclear receptor superfamily members also contain a conserved C-terminal ligand-binding domain (LBD) that is less conserved than the defining DBD. The variable LBD region of nuclear receptor proteins contains a ligand-binding pocket and a dimerization domain, plus regions that interact with cofactors serving as transcriptional intermediary factors (Moras & Gronemeyer, 1998).

Many nuclear receptors regulate transcription via transduction of signals from small, lipophilic molecules such as steroid hormones (Carson-Jurica *et al*., 1990; Tsai & O'Malley, 1994). Other nuclear receptor family members, identified on the basis of DBD homology, do not (or did not when they were initially described) have known ligands. They are therefore referred to as orphan receptors (Giguere, 1999). Phylogenetic analysis demonstrates that the earliest members of the nuclear receptor family were the so-called orphans and that ligand-binding capacity was acquired later in animal evolution (Escriva *et al*., 1997; Bertrand *et al*., 2004).

The 49 nuclear receptors identified in the human genome include receptors for the steroid hormones, thyroid hormones, Vitamin D, and retinoic acid in addition to orphans such as COUP-FT1, *HNF-4* A, and PNR (Robinson-Rechavi *et al*., 2001). By comparison, the genome of the nematode *Caenorhabditis elegans* contains more than 250 nuclear receptors, few of which have known functions or human homologs (Sluder & Maina, 2001). The well-characterized genome of the insect *Drosophila melanogaster*, however, encodes only 18 canonical nuclear receptors, plus 3 additional receptors not found in humans that have the characteristic DNA-binding domain of nuclear receptors but lack the LBD (the knirps family; Adams *et al*., 2000).

With the exception of the small knirps family, all of the Drosophila nuclear receptors have been matched with human orthologs (King-Jones & Thummel, 2005). This finding strongly supports the phylogenetic hypothesis that all subfamilies of nuclear receptors were present in metazoans prior to the split of the protostome and deuterostome lineages (Laudet, 1997; Bertrand *et al*., 2004). The genome of the malaria mosquito *Anopheles gambiae*, another representative of the dipteran insect lineage, contains an identical set of 18 canonical nuclear receptors, although one member of the knirps family appears to be absent from the mosquito genome (Holt *et al*., 2002; Bertrand *et al*., 2004).

The sequencing of the honey bee genome by the Baylor Human Genome Sequencing Center and the full public availability of these sequences offers the first opportunity for a detailed bioinformatics analysis of the nuclear receptor superfamily in a non-dipteran insect. The fly/bee comparison is of extraordinary interest for many reasons. Given that the time since divergence of the Drosophila and Apis lineages is measured in hundreds of millions of years, the identification of matched orthologous nuclear receptors in the two genomes should reveal the fundamental set of nuclear receptors required to ‘make’ an endopterygote insect (Robertson, 2005). This leads to the prediction that the nuclear receptors encoded in the bee genome will have significant overlap with those identified in the Drosophila genome. Another possibility is that the honey bee genome might encode additional insect nuclear receptors not present in the Drosophila genome. This prediction is based on the finding that a large scale bee brain expressed sequence tag (EST) project revealed about 100 genes that bees share with humans but that appear to have been lost from the Drosophila lineage (Whitfield *et al*., 2002; Velarde *et al*., 2005).

Why might the nuclear receptors of the bee differ from those of the fly? The behavioural ecology of two insect species could hardly be more different than that *of D. melanogaster* and *A. mellifera*. Drosophilids are small fruit flies that feed individually on rotting fruit; honey bees are obligately social, tree-nesting pollen and nectar feeders that live in large colonies consisting of tens of thousands of individuals (Winston, 1987). Bees navigate accurately over long distances using a sun compass and share information about the location of resources by means of a symbolic language. Colonies are characterized by the presence of two distinct female castes (reproductive queens and sterile workers) and by division of labour on the basis of worker age. This extended process of behavioural development after the completion of metamorphosis is common to many social insects, and is referred to as age polyethism. Age polyethism takes the following form in the bee: workers perform several different tasks in the hive during the first 2–3 weeks of adult life, including comb construction and brood care, and then shift to taking daily foraging trips outside the hive for the remainder of their 5- to 7-week life (Robinson, 1992). The transition to foraging in the honey bee involves long-term, environmentally modulated changes in behaviour that are associated with changes in brain structure, endocrine activity, neurochemistry, and brain gene expression (Withers *et al*., 1993; Robinson, 2002; Whitfield *et al*., 2003). We speculated that the secrets of the evolution of insect eusociality might be at least partly explained in terms of diversification of nuclear receptors. This speculation was fuelled by findings implicating juvenile hormone (JH) and ecdysteroids, along with several of the previously characterized bee nuclear receptors (*AmEcR*, *AmUSP*) known to interact with these hormones, in the regulation of both reproductive and behavioural division of labour in bees and other hymenopterans (Nijhout, 1994; Hartfelder & Engels, 1998; Bloch *et al.*, 2002).

In addition to contributing to our knowledge of the evolution of nuclear receptors in insects, a major motivation for this annotation project was to provide the foundation for the development of new tools for postgenomic functional analyses, particularly of the adult bee brain. The primary uses of the honey bee for probing brain-behaviour relationships have been in studies of learning in the behavioural transition to foraging in adults, while the vast majority of the functional annotation of nuclear receptors in Drosophila involves signalling pathways activated during embryogenesis and/or metamorphosis, but not in adults (King-Jones & Thummel, 2005). Nuclear receptors known to be expressed in the ovaries of adult female flies (*EcR*, *USP*, *Eip75B*) constitute the rare exceptions to this generalization. Characterization of the nuclear receptors of the bee allows the study of these transcription factors in contexts other than early development or reproduction, and permits an examination of the role of nuclear receptors in the development of adult-specific behaviours such as foraging and the regulation of structural plasticity in the adult brain (Robinson, 2002; Fahrbach, 2006).

The nuclear receptors of the bee were poorly characterized prior to the availability of the sequenced genome. A cDNA (AF263459) encoding the Apis ortholog of ultraspiracle (USP) was cloned and sequenced and shown to be expressed in many tissues of the adult worker and queen (Barchuk *et al*., 2004). An unpublished partial sequence for Apis ecdysone receptor (EcR) is available in GenBank (AB095514). Our analysis of EST sequences from a project based on adult honey bee brain (http://titan.biotec.uiuc.edu/bee/honeybee_project.htm) provided evidence for the expression of at least 5 nuclear receptors in adult brain: *AmUSP*, *AmSVP*, *AmHnf-4*, AmERR, and *AmHr46*. An additional EST encoding fushi tarazu-factor 1 (Ftz-F1) was identified in a study of gene expression in the brain during adult behavioural development (Kucharski & Maleszka, 2002; Bertrand *et al*., 2004). These exceedingly sparse data have prevented any generalizations about the nuclear receptor family in any insect order other than the Diptera and blocked any comparisons of the nuclear receptors of the fly and the bee.

This paper presents the first genome-based overview of nuclear receptors in *Ap. mellifera*. For clarity, we refer to the previously annotated members of the nuclear receptor superfamily identified in the genome of D. melanogaster with the prefix ‘Dm’, and give the nuclear receptors identified in the genome of *A. mellifera* the prefix ‘Am.’

## Results

### *Identification of nuclear receptors in the genome of* Apis mellifera

We used all available sequence information for *A. mellifera* (versions 2.0 and 3.0) and all gene prediction sets (Ensembl, Gnomen, Heidelberg, Eisen, FgenesH, GLEAN3) to identify nuclear receptors in the honey bee genome. The conserved modular organization of nuclear receptors greatly facilitates Blast-based, in silico detection of nuclear receptor sequences, and we are confident that our analysis is complete (Robinson-Rechavi & Laudet, 2003). Table 1 presents the Apis ortholog for each of the previously annotated Drosophila nuclear receptors. To facilitate comparison, we have used the names of the fly receptors to name the bee receptors. The diversity of known nuclear receptors has been organized into a database (NuReBASE) in which nuclear receptors are systematically named based on phylogenetic interpretations (Nuclear Receptors Committee, 1999). Table 1 gives our NuReBASE nomenclature for each of the Apis nuclear receptors. The only previously known insect nuclear receptor that could not be completely recovered from the available assembled Apis genome sequence was hepatocyte nuclear factor-4 (*AmHnf-4*). However, a cDNA encoding a presumed Apis *Hnf-4* was previously recovered from an Apis EST project (Whitfield *et al*., 2002; Bertrand *et al*., 2004), and it was possible to reconstruct the DBD and LBD from unassembled genome sequences. We are therefore confident that *Hnf-4* is present in the honey bee genome.

**Table 1:** Nuclear receptors of *Apis mellifera*.

NuReBASE Nomenclature1	Drosophila Receptor2	Apis Ortholog3	Am/Dm DBD % Identity	Am/Dm LBD % Identity	Apis Accession Number
NR0A1	Knirps (*DmKnr*) FBgn0001320	*AmKnr*	82	*	GB15945
NR0A2	Knirps-like (*DmKnr*l) FBgn0001323	*AmKnr*l	91	*	GB13710
NR0A3	Eagle (DmEg) FBgn0000560	*AmEg*	86	*	GB18215
NR1D3	Ecdysone-induced protein 75B (*DmEip*75B) FBgn0000568	*AmEip75B*	100	60	GB11364
NR1E1	Ecdysone-induced protein 78C (*DmEip*78C) FBgn0004865	*AmEip78C*	89	58	GB30226
NR1F4	Hormone receptor-like in 46 (*DmHr46*) FBgn0000448	*AmHr46*	97	65	GB10650
NR1H1	Ecdysone receptor (*DmEcR*) FBgn0000546	*AmEcR*	88	66	GB30298
NR1JI	Hormone receptor-like in 96 (*DmHr96*) FBgn0015240	*AmHr96*	76	69	GB10331
NR2A4	Hepatocyte nuclear factor 4 (*DmHnf4*) FBgn0004914	*AmHnf4*	91	75	GB11424
NR2B4	Ultraspiracle (DmUSP) FBgn0003964	*AmUSP*	91	47	GB16648
NR2D1	Hormone receptor-like in 78 (*DmHr78*) FBgn0015239	*AmHr78*	92	31	GB18358
NR2E1	Tailless (*DmTll*) FBgn0003720	*AmTll*	82	35	GB20053
NR2E3	Hormone receptor-like in 51 (*DmHr51*) FBgn0034012	*AmHr51*	98	61	GB10077
NR2E4	Dissatisfaction (*DmDsf*) FBgn0015381	*AmDsf*	83	56	GB14217
NR2E5	Hormone receptor-like in 83 (*DmHr83*) FBgn0037436	*AmHr83*	73	27	GB17656
NR2E6 (proposed)	**	*AmPNR*-like (proposed)	61^4^	45^4^	GB17775
NR2F1	Seven up (*DmSVP*) FBgn0003651	*AmSVP*	95	98	GB17100
NR3B4	Estrogen-related receptor (*DmERR*) FBgn0035849	*AmERR*	95	55	GB11125
NR4A1	Hormone receptor-like in 38 (*DmHr38*) FBgn0014859	*AmHr38*	95	74	GB17814
NR5A3	Ftz transcription factor 1(*DmFtz-F1*) FBgn0001078	*AmFtz-F1*	100	75	GB16873
NR5B1	Hormone receptor-like in 39 (*DmHr39*) FBgn0010229	*AmHr39*	86	79	GB11634
NR6A2	Hormone receptor-like in 4 (*DmHr4*) FBgn0023546	*AmHr4*	96	51	GB16863

1 Nuclear Receptors Nomenclature Committee 1999.

2 Drosophila receptors are named on the basis of mutant phenotype (e.g. tailless), the name given to the orthologous vertebrate receptor (e.g. Estrogen-related receptor), or cytogenetic location in the polytene chromosomes (e.g. Hr83), fly base ID provided.

3 Apis receptors are named in this publication for their apparent Drosophila ortholog with the exception of *AmPNR*-like, which has not been annotated in the Drosophila melanogaster genome.

4 Similarity of *AmPNR*-like was highest to DmUSP at the DBD and to *DmHr51* at the LBD; these values are shown.

* The members of the knirps family have a conserved DBD, but no LBD.

** Not present in Drosophila sequence databases.

In addition to identifying and/or reconstructing orthologs for each of the Drosophila receptors, we also identified a nuclear receptor not present in any Drosophila sequence database. This receptor has relatively high sequence similarity to the photoreceptor-specific nuclear receptor (PNR) of vertebrates, and will be discussed in detail in a subsequent section.

We compared the percentage identity of the DBD and LBD for each of the receptors across Drosophila and Apis. As would be predicted on the basis of past analyses of nuclear receptors, the conservation of the DBDs was high (from 82 to 100% identity), while the LBDs were more divergent (from 27 to 98% identity). Sequence alignments for the Apis receptors (with the exception of *AmHnf-4*) with other known insect and vertebrate sequences have been deposited in GenBank and are presented as online supplemental material accompanying this paper.

### AmPNR-*like is a new member of the* NR2 *subfamily*

The *NR2* subfamily of nuclear receptors contains the human TLL and PNR nuclear receptors and their Drosophila orthologs (*tailless* and the two PNR homologs, *DmHr51* and *DmHr83*), plus *dissatisfaction* (a duplication of *tailless* that occurred after the divergence of the insect and vertebrate lineages) and *C. elegans Fax-1*. A nuclear receptor identified in the anthozoan cnidarian *Acropora millepora* (a coral) is also a member of the *NR2*E group, implying an extremely ancient origin for the group (Grasso *et al*., 2001). On the basis of sequence similarity and phylogenetic analysis, we have identified (and correspondingly named) Apis orthologs for Drosophila *tailless*, *dissatisfaction*, *DmHr51*, and *DmHr83*.

**Figure 1:**
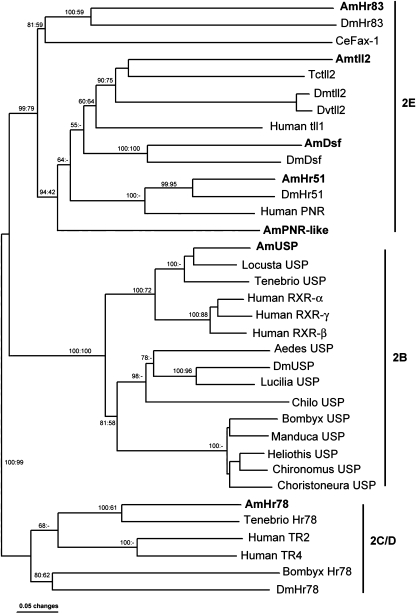
Phylogenetic tree of the *NR2* (*NR2*E, *NR2*B, and *NR2*C/D) nuclear receptor subfamily members showing the clustering of the novel *AmPNR*-like gene within the *NR2*E group. Novel Apis members of these groups identified in our analysis are highlighted in bold. The *AmUSP* phylogenetic relationships were confirmed as previously reported (Barchuk *et al*., 2004). The different groups within *NR2* are indicated to the right. Support for the major branches is indicated as percentage of 1000 bootstrap replicates of neighbour joining and 1000 bootstrap replicates of heuristic parsimony analysis. The human sequences were chosen to represent the vertebrate nuclear receptors; *Drosophila melanogaster* (Dm) sequences and other representative insect sequences were used as available for each group. Sequences used for construction of the tree are given in supplementary material.

Our Blast searches also recovered an additional member of this group from the Apis genome, a gene now given the trivial name *AmPNR*-like. Different chromosomal locations for *AmPNR*-like (Group 12), AmHr51 (Group 1), and AmHr83 (Group 10) initially suggested that this putative novel gene did not represent a misidentification as a consequence of an error in the assembly. Our subsequent phylogenetic analysis clearly identifies *AmPNR*-like as a novel member of the *NR2* subfamily (Fig. 1). Phylogenetic reconstructions with sequences corresponding to the DBD, LBD, and using complete sites of aligned sequences, supported the positioning of *AmPNR*-like in the *NR2*E group as well as the positioning the other identified honey bee nuclear receptors in their expected clades, as shown for the *NR2*E, B, and C/D groups. All previously described members of the *NR2* subfamily are represented in the honey bee genome and cluster with their corresponding insect and vertebrate orthologs.

The lack of any known *AmPNR*-like vertebrate orthologs and its branching at the base of the monophyletic group containing *Hr51*, *Dsf* and *tll* suggest that *AmPNR*-like was present in the common ancestor of bilaterians (Urbilateria) and was subsequently lost from the nematode, dipteran, and vertebrate lineages. Establishing the presence of *AmPNR*-like orthologs in other insect genomes as well as in lophotrochozoans (trematodes and mollusks) will provide crucial support for this hypothesis. Our initial examination of available sequences from the *Tribolium castaneum* (red flour beetle) genome project (http://www.hgsc.bcm.tmc.edu/projects/tribolium/) indicates that an ortholog of *AmPNR*-like is present in the beetle genome (Velarde, unpublished).

**Figure 2:**
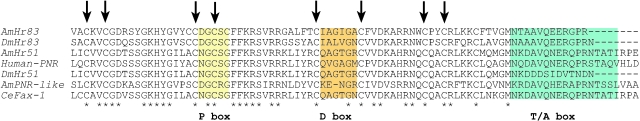
Alignment of the DNA binding domain of *NR2*E members most similar to *AmPNR*-like. Asterisks indicate amino acids conserved in all sequences shown. Arrows indicate the eight zinc-coordinating cysteines conserved in all nuclear receptor DBDs (four per zinc-binding module) Colored boxes indicate the P box (yellow), D box (orange) and T/A box (green).

We also considered the other distinctive features of nuclear receptors in our analysis of *AmPNR*-like. Human PNR has a distinctive proximal box (P box) amino acid sequence of NGCSG associated with the first zinc finger in its DBD (Kobayashi *et al*., 1999). The P box of nuclear receptors is responsible for the interactions of nuclear receptors with specific hormone response elements in DNA upstream of target genes (Hsieh *et al*., 2003). The PNR-type P box sequence of NGCSG is conserved in *AmHr51*, but not in *AmHr83* (Fig. 2). In *AmHr83*, the first asparagine has been replaced by the more common aspartic acid. Although this is a conservative replacement, this suggests that our phylogenetic analysis is correct in clustering the insect *Hr51* genes with the vertebrate PNR genes. By contrast, the P box sequence of *AmPNR*-like has a distinctive replacement in the third position of the P box. Here, the *NR2*E-typical serine or alanine has been replaced by an arginine (Fig. 2). Possibly this unusual P box (DGCRG) targets genes not previously known to be regulated by nuclear receptors. The Distal box (D box), the amino acids between the first and second cysteine of the second zinc figure, is also highlighted in Fig. 2. Note that the D box of *AmPNR*-like is shorter by one amino acid than that of any other closely related nuclear receptor. The D box is important for DNA-binding-mediated dimerization, and this difference also suggests altered function of this group member. The C-terminal extension of the DBD, referred to as the T/A box, is divergent in all of the group members, including *AmPNR*-like (Fig. 2). The molecular relevance of the differences in these motifs among the group members is unknown.

**Figure 3:**
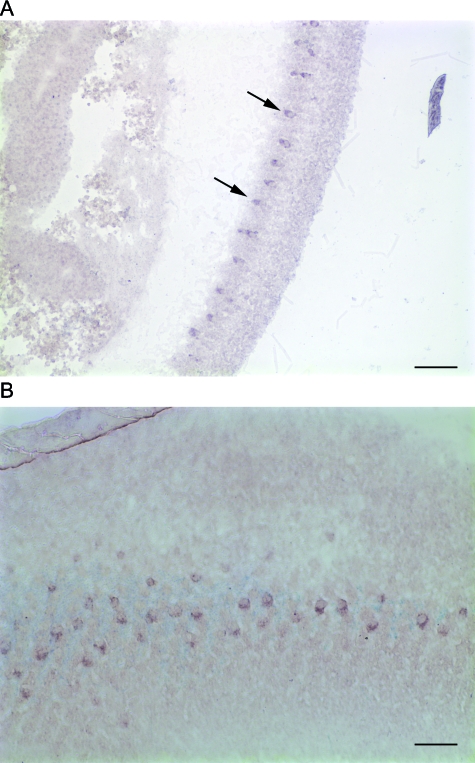
Iocalization of mRNA encoding *AmPNR*-like in the pupal head. A. Transverse section of a developing compound eye hybridized with probe for *AmPNR*-like (arrows); the most intense signal is restricted to a row of large cells along the proximal portion of the retina. The developing optic lobe is visible to the left. Scale bar = 100 m. B. Sagittal section hybridized with the same probe as in A. Scale bar = 50 m.

We have addressed the possibility that *AmPNR*-like is a pseudogene. Pseudogene nuclear receptors have not been described in insects, but have been previously identified in the human genome and may also account in part for the exceptionally large number of nuclear receptors identified through bioinformatics in the *C. elegans* genome (Hodgkin, 2001; Maglich *et al.*, 2001; Robinson-Rechavi *et al.*, 2001). We therefore sought evidence for the expression of *AmPNR*-like in bee tissues. Both reverse transcriptase polymerase chain reaction (data not shown) and *in situ* hybridization studies revealed the presence of detectable transcript in early pupal tissue samples that contained the brain and developing compound eyes (Fig. 3). In tissue sections, intense expression of *AmPNR*-like transcript was evident in a small number of cells in the developing eyes, although without the use of additional markers not yet available for the bee it is not possible to state if these cells are photoreceptors or support cells (Fig. 3). The general pattern, however, is strikingly similar to the highly restricted pattern of retinal expression reported for the transcript of the human PNR gene (Kobayashi *et al.*, 1999). Taken together with the absence of stop codons in the coding regions of the gene, this evidence indicates that *AmPNR*-like is not likely to be a pseudogene. It is not absolutely definitive because some mammalian pseudogene nuclear receptors such as FXR-r have been reported to be transcribed (Maglich *et al.*, 2001).

### *Modulation of* AmUSP *expression in the adult bee brain during behavioural development*

The Drosophila ultraspiracle (USP) locus was initially identified in an analysis of recessive lethal mutations representing genes within the 2C-D area of the X chromosome; offspring of heterozygotic usp/+mothers developed extra posterior spiracles (Perrimon *et al*., 1985). Subsequent studies in insects and other arthropods demonstrated that the protein encoded by USP heterodimerizes with the product of EcR to form the functional receptor for the insect steroid hormone 20-hydroxyecdysone (Yao *et al*., 1993). The USP nuclear receptor is orthologous to the vertebrate retinoid X receptor (RXR), which binds the ligand 9-*cis*-retinoic acid (Oro *et al*., 1990). Analysis of the USP LBD, however, has revealed that the invertebrate receptor cannot bind retinoic acid (Clayton *et al*., 2001), and USP is currently designated an orphan receptor. Studies in Drosophila have revealed that USP can dimerize with nuclear receptors other than EcR, including SVP and *DmHr38* (Sutherland *et al*., 1995; Zelhof *et al*., 1995), indicating that this receptor functions in multiple regulatory pathways during development.

**Figure 4:**
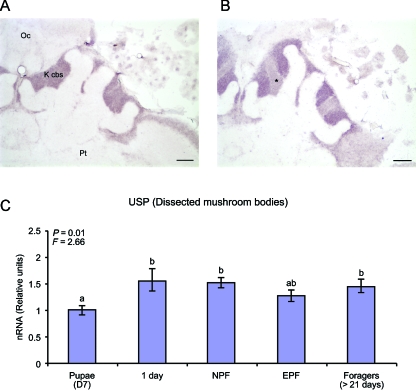
Localization and relative quantification of *AmUSP* mRNA in mushroom bodies of the bee brain. A. The mushroom bodies are seen in transverse section through the brain of a 1 day old hybridized with probe for *AmUSP*. B. Mushroom bodies of a > 21 day old forager brain hybridized with the same probe as A. Asterisk indicates the region of the inner compact Kenyon cells. K cbs, Kenyon cell bodies; Oc, ocelli; Pt, protocerebrum. Scale bars = 100 µm. C. qRT-PCR analysis of *AmUSP* expression in individual mushroom bodies of 7 day pupae, 1 day workers, new precocious foragers (NPF), experienced precocious foragers (EPF), and foragers older than 21 days (2 genotypes; *N* = 8 brains per group). Data are means ± SE (converted as relative units to the lowest group mean). P and * F*-values for a one way anova are indicated. Bars with the same letter indicate means are not significantly different (*t*-test for LSD *P* > 0.05).

We identified a cDNA encoding *AmUSP* from a bee brain-based EST project (http://titan.biotec.uiuc.edu/bee/honeybee_project.htm), which indicated that this nuclear receptor is expressed in the adult bee brain. We therefore used quantitative RT-PCR (qRT-PCR) and *in situ* hybridization, with the complete sequence of this transcript, to define the expression of USP mRNA in the adult bee brain in relationship to adult worker behavioural development. *AmUSP* is expressed in specific cell populations of the adult honey bee brain, including the Kenyon cells, the intrinsic neurones of the protocerebral mushroom bodies, an insect brain region critical for learning and memory (Fahrbach, 2006). Kenyon cells can be divided into distinct subpopulations that reflect earlier and later birth dates during postembryonic neurogenesis. A central ‘core’, designated the inner compact cells in the honey bee, represents the last-born subpopulation of Kenyon cells (Farris *et al*., 1999). qRT-PCR measurements from dissected whole mushroom bodies revealed that expression of *AmUSP* is maintained in the mushroom body neurones during the entire course of adult behavioural development, in both normal age (Foragers > 21 days) and younger foragers (NPF and EPF) (Fig. 4C). Our *in situ* hybridization studies (Fig. 4.A,B) provided a finer grained analysis, revealing a relative decrease in expression of *AmUSP* transcript specific to the inner compact Kenyon cells when 1 day old worker honey bees (Fig. 4A) were compared with old foragers (Fig. 4B).

### Modulation of AmSVP expression in the adult bee brain during behavioural development

The chicken ovalbumin upstream promoter-transcription-factors (*COUP-TF*) group of nuclear receptors are orphans expressed in specific neuronal cell lineages in all animals studied, including cnidarians such as the hydra (Broadus & Doe, 1995; Gauchat *et al*., 2004). The Drosophila *COUP-TF* gene is named seven-up (SVP) for its critical role in the specification of photoreceptor cell identity during the formation of the fly compound eye during metamorphosis; in SVP mutants, photoreceptors designated R1, R3, R4, and R6 switch their phenotype to that of R7 (Mlodzik *et al*., 1990). More recently, a role for SVP in the development of non-neural insect tissues, notably the Malpighian tubules (insect kidneys) and the heart, has also been defined (Kerber *et al*., 1998; Lo & Frasch, 2001).

**Figure 5:**
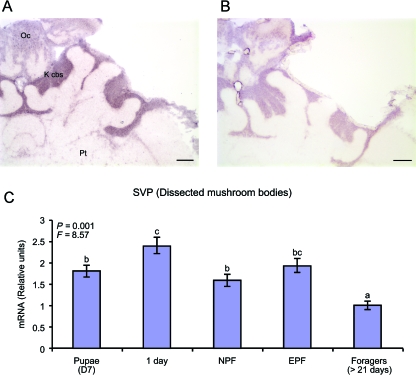
Localization and relative quantification of *AmSVP* mRNA in mushroom bodies of the bee brain. A. Mushroom bodies corresponding to a transverse section of a 1 day old worker brain section hybridized with probe for *AmSVP*. B. Mushroom bodies of a > 21 day old forager brain hybridized with the same probe as A. K cbs, Kenyon cell bodies; Oc, ocelli; Pt, protocerebrum. Scale bars = 100 µm. C. qRT-PCR analysis of *AmSVP* expression in individual mushroom bodies of 7 day pupae, 1 day workers, new precocious foragers (NPF), experienced precocious foragers (EPF), and foragers older than 21 days (2 genotypes; *N* = 8 brains per group). Data are means ± SE (converted as relative units to the lowest group mean). P and *F*-values for a one way anova are indicated. Bars with the same letter indicate means are not significantly different (*t*-test for LSD *P* > 0.05).

We identified a cDNA encoding *AmSVP* from the bee brain EST project previously described, and qRT-PCR and *in situ* hybridization were used to define its expression in the adult bee brain (Fig. 5). qRT-PCR analysis of *AmSVP* expression in dissected mushroom bodies of bees sampled at various stages during the development of foraging suggested that *AmSVP* expression is down-regulated in the mushroom bodies of foragers relative to 1 day old bees, with the lowest expression observed in the oldest, most-experienced foragers (Foragers > 21 days) (Fig. 5C). *In situ* hybridization analysis revealed that *AmSVP* is expressed abundantly by all subpopulations of Kenyon cells in the mushroom bodies of 1 day old adult worker bees (Fig. 5A). Its expression is reduced in the mushroom bodies for foragers (Fig. 5B).

## Discussion

### Conservation of nuclear receptors in insects

All of the Drosophila nuclear receptors were found to be in the published genome of the honey bee, *A. mellifera*, a match that suggests the Drosophila set will be conserved across the Insecta. This extraordinary conservation, across *c*. 300 million years of evolution, strongly suggests that the Drosophila set constitutes the essential set of insect nuclear receptors. This speculation awaits the results of further insect genome sequencing projects and detailed functional analyses.

This conservation implies that functional analyses of nuclear receptor function in different insect species will have broadly applicable findings. Our analysis does not support the hypothesis of extensive gene losses in Drosophila relative to other insect lineages, nor does it support the view that evolution of insect sociality is associated with diversification of nuclear receptors. It should be noted that one aspect of nuclear receptors beyond the reach of genome analysis is the number of isoforms that can be produced from each gene via alternative splice sites and alternative promoters. This requires extensive characterization of cDNAs and the proteins they encode. Multiple isoforms are known for many of the Drosophila nuclear receptors, resulting in a nuclear receptor proteome that is significantly larger than the relatively small number of nuclear receptor genes encoded in the genome (King-Jones & Thummel, 2005). Now that the comparison of the fly and bee genomes is completed, the important and more difficult task of defining the population of functional nuclear receptors in the bee can begin.

Many functional studies of nuclear receptors will be most efficiently conducted in Drosophila; this work has already begun (King-Jones & Thummel, 2005). Such studies will enhance our knowledge of transcriptional regulation via nuclear receptors as well as our knowledge of insect development and physiology. But there are specific aspects of function regulated by nuclear receptors that cannot be studied in Drosophila. The clearest example of the limits of the fruit fly as a model for understanding the physiology of the social Hymenoptera is seen when two key aspects of sociality in the honey bee are considered: caste determination and division of labour on the basis of worker age. Endocrine signalling via nuclear receptors has been repeatedly implicated in regulating the expression of these polymorphisms (queens and workers) and polyphenisms (hive bees and forager bees) (Bloch *et al*., 2002). The striking conservation of fly nuclear receptors in the honey bee indicates that, if nuclear receptors regulate caste and behavioural polyphenisms in the social Hymenoptera, they do so through altered patterns of transcriptional regulation and endocrine secretion rather than via expansion of the nuclear receptor superfamily.

The single novelty uncovered in our analysis is the presence in the Apis genome of a third nuclear hormone gene homologous to human PNR. Two putative orthologs of PNR designated *DmHr51* and *DmHr83* have been previously reported for Drosophila (King-Jones & Thummel, 2005). The Apis genome contains sequences with high homology to *DmHr51* and *DmHr83*, but also contains an additional member of the *NR2* subfamily, *AmPNR*-like.

### Expression of AmUSP in the adult bee brain

Our analyses of sequences from a bee brain EST project and experiments with quantitative RT-PCR and *in situ* hybridization demonstrate that *AmUSP* mRNA is expressed in the adult honey bee brain, most notably within the neurones of the mushroom bodies. This is consistent with a previous report, based on Northern blots, that mRNA encoding *AmUSP* was present in the heads of 24 h honey bee workers (Barchuk *et al*., 2004). We have now provided evidence for *AmUSP* expression across the entire span of bee adult behavioural development and discovered a selective reduction in *AmUSP* expression in the inner compact subpopulation of Kenyon cells in forager bees. The dendritic arborizations of these neurones are longer and more branched in these neurones in foragers than in bees that work exclusively within the hive (Farris *et al*., 2001); the reduction in *AmUSP* expression is temporally correlated with this increase in dendritic complexity.

The reduction in USP gene expression is also temporally correlated with the increased titres of JH associated with foraging (Fahrbach, 1997). JH is a sesquiterpenoid that regulates numerous aspects of development and reproduction in insects (Nijhout, 1994). At present, the USP nuclear receptor has no known ligand, and JH has no known cellular receptor. The possibility that USP's missing ligand could be JH is supported by *in vitro* studies of binding (Jones & Sharp, 1997; Jones *et al*., 2001; Xu *et al*., 2002), although *in vivo* evidence is inconclusive (King-Jones & Thummel, 2005). Modulated expression of the USP gene product by the neurones of the mushroom bodies suggests a mechanism by which JH can coordinate behaviour and brain structure. Because JH titres in worker honey bees are regulated by a combination of social and environmental cues (Huang & Robinson, 1992; Elekonich & Roberts, 2005), detailed comparative studies of USP expression and function in fly and bee brains could reveal, within the context of specific neural circuits, the molecular basis of socially mediated developmental plasticity in the bee. We have focused our attention on the possibility that *AmUSP* functions as a JH receptor because interactions of *AmUSP* with liganded *AmEcR* are unlikely, given that ecdysteroids are not produced by worker bees after the first few days of adult life (Robinson *et al*., 1992; Hartfelder *et al*., 2002). A previous study showed that treatment of worker and queen bees with JH induced a transient up-regulation of *AmUSP* expression in fat body (Barchuk *et al*., 2004). Our data show, in contrast, that the maintenance of the high levels of JH typical of foraging bees is correlated with a reduction of *AmUSP* mRNA expression in specific neuronal populations.

A function of the unliganded EcR/USP complex as a suppressor of gene expression during the early phases of metamorphosis has been previously demonstrated in Drosophila using cultured wing discs (Schubiger & Truman, 2000). Studies of the precocious differentiation of wing sensory neurones in Drosophila in response to reduction in EcR and USP expression also argue in favour of a repressive function for the unliganded receptor complex (Schubiger *et al*., 2005). A role for JH in the relief of suppression through interactions with USP has not yet been reported.

### Expression of AmSVP in the adult bee brain

Our initial characterization of *AmSVP* expression in the adult bee brain was not directly linked to endocrine function, as in the case of a possible relationship of USP with juvenile hormone. Rather, the notable feature here for *AmSVP* is expression in the brain at the adult stage of development, for which no previous literature exists. The Drosophila SVP gene is a member of the *NR2*F subfamily of nuclear receptors that includes the orphan *COUP-TF*s. *COUP-TF*s generally function as negative regulators of transcription, both as homodimers and by forming heterodimers that reduce the availability of other nuclear receptors such as RXR, the vertebrate ortholog of insect USPs (Pereira *et al*., 2000). In all of its previously characterized physiological functions, *DmSVP* exerts its actions through transient expression in localized cell populations. Therefore, our evidence of widespread, persistent expression of *AmSVP* in the brain throughout a prolonged life stage was unexpected. Even more interesting is the reduction in *AmSVP* expression in the mushroom bodies in association with the onset of foraging. Although *COUP-TF*s are also known to function as gene activators (Gaudet & Ginsburg, 1995), the simplest model suggests that the demonstrated reduction in *AmSVP* expression results in derepression of genes related to the function of mushroom body neurones in the foraging bee. Because foraging experience induces the growth of dendrites of mushroom body neurones, genes associated with dendritic extension and branching are among the candidates for genes released from repression by reduced *AmSVP* expression (Farris *et al*., 2001). An alternative, not mutually exclusive model is that reduction in *AmSVP* expression in foraging bees frees USP receptors to dimerize with other partners within the mushroom body neurones, or to respond to juvenile hormone. These predictions can be investigated in cultures of primary cultures of Kenyon cells.

### What is AmPNR-like?

Our phylogenetic analyses suggest that *AmPNR*-like represents a novel member of the *NR2* subfamily that has been lost from the Drosophila, *C. elegans*, and human genomes. The branching of *AmPNR*-like at the base of the *tll/PNR* group and the lack of vertebrate orthologs supports its possible ancestral nature and clearly eliminates the possibility of an origin from a recent gene duplication. Phylogenetic reconstructions and genome-wide analysis for the nuclear receptor family suggest that most gene duplication or loss occurred very early in animal evolution (approximately 400 million years ago), and that early gene loss can largely explain the characteristic patterns observed in the nuclear receptor profiles of different lineages (Bertrand *et al*., 2004). In this regard, two clusters within the *NR2*E group (*Hr83* and *Fax:1*; *Dsf*, *tll*, and *Hr51*) are considered early losses from the chordate and nematode lineages, respectively. In contrast, all are represented in the insect lineage, supporting the hypothesis that *AmPNR*-like was retained in the early arthropods and subsequently lost from the dipteran lineage. The prediction that orthologs of *AmPNR*-like should also be discovered in the genomes of other insects will be testable in the near future (Robertson, 2005) and, together with the availability of lophotrochozoan sequences, will help determine the *AmPNR*-like ancestral gene state.

Mammalian PNRs are notable for a pattern of expression restricted to photoreceptor cells of the retina (Kobayashi *et al*., 1999). We examined the expression of *AmPNR*-like mRNA in sections through the brain and compound eye of pupal and adult worker honey bees using *in situ* hybridization. These studies revealed that this gene is intensely expressed in a regular array of cells in the developing compound eye. If *DmHr51* and *DmHr83* regulate eye development, as suggested by their phylogenetic relationship to mammalian PNRs, the presence of an additional member of the *NR2*E group in the honey bee genome raises many interesting questions.

## Experimental procedures

### Identification of genes encoding nuclear receptors

A set of 21 nuclear receptor proteins from *D. melanogaster* (the 18 canonical receptors plus the knirps family) was used to query the honey bee genome datasets to find putative honey bee nuclear receptors. In cases where there are multiple known isoforms of the Drosophila receptors, the longest one was chosen as the query. All queries were performed against the BeeBase datasets, which included a number of gene prediction sets prepared for the honey bee genome version 2 assembly (http://racerx00.tamu.edu/bee_resources.html) as well as a combined prediction data set (Glean3). Initially, each single Drosophila protein was queried against the predicted protein dataset by BLASTP (Altschul *et al.*, 1990). All hits with an *e*-value < 10–4 were used to search the Drosophila proteome (http://flybase.bio.indiana.edu/) for preliminary homolog identification. These new bee sequences were then examined manually, first by searching for their genomic location using TBLASTN in BeeBase with the ‘Scaffolds-assembly 2’ and the ‘Chromosomes assembly 2’ datasets (Altschul *et al.*, 1990). In addition, TBLASTN was used to identify putative nuclear receptor sequences that were not included in the combined prediction data set or were present in the unassembled sequences (bin 0 reads assembly 2 dataset). The resulting hits provided a link to the genome browser that also contained a map of the different gene predictions. After finding the region encoding a candidate nuclear receptor, two approaches were followed to determine if the predictions were correct, and to create a corrected model RNA if necessary. First, the different predictions were aligned with all insect members and selected vertebrate members of the particular nuclear receptor subfamily (sequences obtained from NuReBASE and NCBI) by CLUSTALX; minor editing of the alignments was performed with JalView (Duarte *et al.*, 2002; http://www.Jalview.org). Second, major alignment discrepancies were used as feedback information to return to the gene sequence and manually check for potential missing exons, erroneous splice site predictions, truncations, frameshifts and erroneous stop codons. The manual editing, as well as the alignment information, were then used to construct a corrected model RNA that was checked in ORFinder as encoding a putative nuclear receptor with a complete DBD and LBD (with the exception of knirps family members, which lack any LBD). Additional checks for proper identification were performed by aligning the corresponding DNA sequence with existing ESTs or full length cDNAs in the rare cases they were available. Finally, all identified nuclear receptors were analysed for consistency in their phylogenetic positioning among known nuclear receptors by neighbour-joining.

### Phylogenetic analysis

Sequences (*N* = 35) representing the diversity of *NR2*E, 2B, and C/D subfamily proteins were aligned using CLUSTALX (Jeanmougin *et al*., 1998; Hall, 2001). The multiple sequence alignments were then inspected and manually refined with JalView (Clamp *et al*., 2004). Only complete sites were used for the phylogenetic analysis. Phylogenetic tree reconstruction was performed using the minimum distance heuristic algorithm in paup* v4.0b10 (Swofford, 2003).

### Animals

Bees were collected for qRT-PCR and *in situ* hybridization studies from colonies maintained at the Bee Research Facility of the University of Illinois at Urbana-Champaign (Urbana, IL). All brood frames used in each experiment were obtained from the same single drone inseminated (SDI) source colony. To obtain pupae at 7 days of development (2 days prior to emergence) and 1 day old adult bees we followed published procedures (Farris *et al*., 2001). Bees of the same age (11 days) but with different amounts of foraging experience were collected from a small single cohort colony (Robinson *et al*., 1989): single cohort colonies stimulate the precocious development of foraging and allow the uncoupling of the behavioural transition to foraging from worker age. Six days after the single cohort colony was established, observations of the hive entrance were made for 1 h in the morning and 1 h in the afternoon; returning marked bees identified as foragers (bees carrying pollen or with a nectar-distended abdomen) were marked on the abdomen. At 11 days of age, foragers marked on the thorax and abdomen were collected and designated experienced precocious foragers (EPF). The same day, returning foragers with only a thoracic mark were collected and designated new precocious foragers (NPF), as the lack of paint on the abdomen indicated that they had made the transition to foraging that day. Experienced foraging bees > 21 days old were obtained by marking 500 1 day old bees and re-introducing them to the source colony. These bees were collected at the colony entrance 21–25 days later.

### Quantitative Real Time-PCR

Bees for qRT-PCR studies were collected into liquid nitrogen and decapitated on dry ice. Heads were stored at −80 °C until dissection. Bee heads were partially lyophilized to facilitate brain dissection (Schulz & Robinson, 1999). Removal of the brains from the head capsule was performed in dissecting dishes placed on a bed of dry ice. The mushroom bodies were dissected from each brain on dry ice under a 100% ethanol bath. Total RNA was isolated from the dissected mushroom bodies of each individual bee with the Rneasy mini kit (Qiagen Valencia, CA). We quantified *AmSVP* and *AmUSP* mRNA with real-time quantitative PCR (qRT-PCR) using an ABI Prism 7900 sequence detection system (Applied Biosystems, Foster City, CA). Specific primers and probes (Taqman) were designed for each gene (Table 1). All qRT-PCR reactions were performed in triplicate. RT-PCR data was analysed using the 2^−ΔΔC^T relative quantification method (Livak & Schmittgen, 2001). The Apis ortholog of the housekeeping gene *rp49* (AF441189) was used as an internal control (Ben-Shahar *et al*., 2002; Grozinger *et al*., 2003; Corona *et al*., 2005). The average amount of *AmUSP* and *AmSVP* mRNA normalized to *rp49* and relative to the lowest group mean is reported. Gene expression levels for each group (pupae, 1 day, NPF, EPF, and Foragers > 21 days) were compared using the General Linear Models procedure (PROC GLM, SAS v 9.1, SAS Institute, 2004).

### *In situ* hybridization

Probes for *in situ* hybridization were prepared for each nuclear receptor to be localized from PCR products using specific primers with T3 and T7 promoters attached to the 5′-and 3′ primers, respectively (see Table). Synthesis of riboprobes and digoxigenin-labelling were performed by means of *in vitro* transcription using Roche RNA Labeling Mix (Roche 1277073, Indianapolis, IN). Probes were 400–700 bases in length. Brains to be used in hybridization studies were dissected from the head capsule of cold-anaesthetized bees in a small drop of bee saline (Huang *et al*., 1991). Dissected brains and/or whole pupae were immediately transferred to Cryo-M-Bed embedding compound (Bright Instrument Company Ltd, Huntingdon, UK), frozen on to cryostat chucks using powdered dry ice, sectioned at 10 µm, and thaw-mounted on to FisherPlus slides (Fisher Scientific, Pittsburgh, PA). After overnight air-drying, sections were fixed in 4% paraformaldehyde, deproteinized with proteinase K (Sigma P5568 St Louis, MO), and treated with acetic anhydride prior to hybridization with a digoxigenin-labelled riboprobe (1000 ng/ml) at 50 °C overnight in 50% formamide. Following posthybridization rinses, sections were incubated with a sheep antidigoxigenin-alkaline phosphatase antibody (Roche 1093274), treated with levamisole to block endogenous alkaline phosphatase activity, and developed in NBT/BCIP (Vector Laboratories, Burlington, CA). Developed slides were coverslipped with CrystalMount (Biomeda, Foster City, CA) or glycerol. Sense strand probes were used as controls. All solutions used prior to hybridization were RNase-free. Positive controls for the method and reagents included opsin antisense probes for worker brains (Velarde *et al*., 2005) and antisense vitellogenin probes for queen abdominal sections.

## Supplementary material

S1 Sequences Used in Fasta Format.

S2 CLUSTAL X (1.83) multiple sequence alignment.

This material is available as part of the online article from http://www.blackwell-synergy.com
