# Supplementary material for: Nuclear receptors of the honey bee: annotation and expression in the adult brain
Source: Insect Mol Biol. 2006 Oct 1;15(5):583–95. doi: 10.1111/j.1365-2583.2006.00679.x (PMC1847479; doi:10.1111/j.1365-2583.2006.00679.x)
Supplement: S1 — Sequences Used in Fasta Format. [file imb0015-0583-s1.rtf]

S1. Sequences Used in Fasta Format.

>AmHr83/1-299
MGRTLPTTVACKVCGDRSYGKHYGVYCCDGCSCFFKRSVRRGALFTCIAGIGACFVDKARRNWCPYCRLKKC
FTVGMNTAAVQEERGPRVRTKIAAVARDFFRISSSSPTPLPPPILSTGDTIRYEIAARIFFATVLAARQHRE
FSMLDLEEQNKILRRGWAAAFVLRAAIWPIDLTNFRNTSTSNDDTVNDAIFAARAVISSLQPDRIEFSVLET
LILCRPEIAETMNGIRLTSRAMDTAVEILARHLAGKTESSVRVAKLMLILPILTASCPRKLANDLFAPIIGD
IDLEKVIASVR
>DmHr83/1-278
MSNFSACAVCGDQSSGKHYGVSCCDGCSCFFKRSVRRGSSYACIALVGNCVVDKARRNWCPSCRFQRCLAVG
MNAAAVQEERGPRNQQVALYRTGRRQAPPSQAAPSPTPHSQALHFQILAQILVTCLRQAKANEQFALLDRCQ
QDAIFQVVWSEIFVLRASHWSLDISAMIDGCGDEQLKRLICEAHQLRADVLELNFMESLILCRKELAINAEY
AVILGSHSKAALISLARYTLQQSNYLRFGQLLLGLRQLCLRRFDCALSCMFRSVVRDILKTL
>AmHr51/1-660
MNGATSVTTSEEHQHQHQQPQNDPSQEHPRSPTSPLSIRHDSGESSVISPGLPERYSPLGATGSTSSHDPYA
SRSVQECPVPLCRGIPTDFPLARSPYLTALGNGHPHLLGQVQQQQQQHKPPRSLGLICVVCGDTSSGKHYGI
LACNGCSGFFKRSVRRKLIYRCQAGTGRCVVDKAHRNQCQACRLKKCMQMGMNKDAVQNERQPRNTATIRPE
ALVEMDQERALREAAVAVGVFGCNVEIGGKADLMSLSDLANFLIGSVYRIESSSSMMNPRLRFSDFVSLITE
ATRLFMSALTMAASVLSLLLVFGFFVMLSNIFSNLSKELAFSSSVRFPFIAFNCSSAASRLPRSSASIIPLR
LASSINRSPPVSLAMARYTTPLPLPTVAPTTNSTNPPTPPRQDNEDEFHLELFTRNASEDSIDVTNEEPLTP
QRSGCTQIPPVIPSLYSPASAETVYETSARLLFMAVKWAKNLPSFASLPFRDQVILLEEAWSELFLLNAVQW
CLPLESSPLFNSAELTALTLSPHPHPHSGIHMQTTTGKPSQVAADVRHLHDTLQRYKAVMVDPAEFACMKAI
VLFRPETRGLKDSSQIENLQDQAQVMLGQHARAQQPGSPARFGRLLLLLPLLRTVPASRVELIYFHRTIGNT
PMEKVLCDMYKN
>Human PNR/1-410
METRPTALMSSTVAAAAPAAGAASRKESPGRWGLGEDPTGVSPSLQCRVCGDSSSGKHYGIYACNGCSGFFK
RSVRRRLIYRCQVGAGMCPVDKAHRNQCQACRLKKCLQAGMNQDAVQNERQPRSTAQVHLDSMESNTESRPE
SLVAPPAPAGRSPRGPTPMSAARALGHHFMASLITAETCAKLEPEDADENIDVTSNDPEFPSSPYSSSSPCG
LDSIHETSARLLFMAVKWAKNLPVFSSLPFRDQVILLEEAWSELFLLGAIQWSLPLDSCPLLAPPEASAAGG
AQGRLTLASMETRVLQETISRFRALAVDPTEFACMKALVLFKPETRGLKDPEHVEALQDQSQVMLSQHSKAH
HPSQPVRFGKLLLLLPSLRFITAERIELLFFRKTIGNTPMEKLLCDMFKN
>DmHr51/1-532
MATGRSLLFRVPWYVCLCVCAESAEPGVYWRLRLRLGLPTLAGPHTNTLTLTARTSSCRSIKKERIKASQQA
NAPPELPLKVSVDVNIIIAAHSQRRRIGLVRFHQRESEDRPLAVASPRLQINMEPTAMNPKKLHSPQRHCYT
PPPAPMHGQAPPPTSTGVAPPTQPPPPHPAAPNVPNGRLLSWNHSAAAAAAAAAAQAAANSMNHSSAAEGSS
MTRIKGQNLGLICVVCGDTSSGKHYGILACNGCSGFFKRSVRRKLIYRCQAGTGRCVVDKAHRNQCQACRLK
KCLQMGMNKDDDSIDVTNDNEEPHAVSRSDSSFIMPQFMSPNLYTHQHETVYETSARLLFMAVKWAKNLPSF
ARLSFRDQVILLEESWSELFLLNAIQWCIPLDPTGCALFSVAEHCNNLENNANGDTCITKEELAADVRTLHE
IFCKYKAVLVDPAEFACLKAIVLFRPETRGLKDPAQIENLQDQAHHTKTQFTAQIARFGRLLLMLPLLRMIS
SHKIESIYFQRTIGNTPMEKVLCDMYKN
>AmTll/1-377
MQSQDPQIGHMSVTQQKVPASSSRILYDIPCKVCRDHSSGKHYGIFACDGCAGFFKRSIRRNRQYVCKAKSK
GGCMVDKTHRNQCRACRLAKCIQAGMNKDAVQHERGPRNSTLRRQMALYFKEPEMMANMVPPPAAALDLALP
KPPNEPRVSVPAPAPHHPLPHPVYCNNMAMSKIPVNVAGLPTLPLIPAAITAESICEQAARLLFLNVHWARD
LAVGTNLVIEDQLTLLESSWRELFLLAAAQILPTLDPTPLLPPGPQGLGLAVEVTRFRETLAGFHAMSLDQH
EYACIRAIVLFKAGLDSEPLPSSRSSNGSTSPNTGSRLRDAAAVARLRDGAQLALGQRLSGASFGALSTNVV
SAKLYKYAGYNLSDWNV
>Tctll/1-406
MSEMQSVEGAMVHHLEPHRMQIKPQSPSSSSRILDIPCKVCGDFSSGKHYNIFACDGCAGFFKRSIRRNRQY
VCKAKDEGSCIIDKTHRNQCRACRLKKCQNVGMNKDAVQHERGPRNSTLRRQQMSSYYNESRVMMSPPGNVL
NLTMPKYEPNPSIIDPGPALPPTGFLCNNYPPLPQVPPLPLPPIFPPTMINPSAICESAAQLIFMNVQWVRS
IPAFTCLPLSDQLLLLEESWLDLFVLGAAQFLPLMDFSVLVEACGVLQQEPHRRDAFLKEVADFQETLKKIS
QFQLDAHEFACLRAIVLFKTSFEKPSSSSNQEKTTTESAKISVIQDDAQMRLNKHVTTTYPKQPLRFGKILL
LVSSTFRTISGRTIEDLFFKKVIRDTPIVAIISNMYKNQILGNNNV
>Dmtll/1-452
MQSSEGSPDMMDQKYNSVRLSPAASSRILYHVPCKVCRDHSSGKHYGIYACDGCAGFFKRSIRRSRQYVCKS
QKQGLCVVDKTHRNQCRACRLRKCFEVGMNKDAVQHERGPRNSTLRRHMAMYKDAMMGAGEMPQIPAEILMN
TAALTGFPGVPMPMPGLPQRAGHHPAHMAAFQPPPSAAAVLDLSVPRVPHHPVHQGHHGFFSPTAAYMNALA
TRALPPTPPLMAAEHIKETAAEHLFKNVNWIKSVRAFTELPMPDQLLLLEESWKEFFILAMAQYLMPMNFAQ
LLFVYESENANREIMGMVTREVHAFQEVLNQLCHLNIDSTEYECLRAISLFRKSPPSASSTEDLANSSILTG
SGSPNSSASAESRGLLESGKVAAMHNDARSALHNYIQRTHPSQPMRFQTLLGVVQLMHKVSSFTIEELFFRK
TIGDITIVRLISDMYSQRKI
>Dvtll/1-450
MQSSEGSPDMMDQKYNSVRLSPAASSRILYHVPCKVCRDHSSGKHYGIYACDGCAGFFKRSIRRSRQYVCKS
QKQGLCVVDKTHRNQCRACRLRKCFEVGMNKDAVQHERGPRNSTLRRHMAMYKDAMMGAAEMPQIPPEILMN
TAALTGFPGLPMPMPGVQRSHHHAALSAAFQPPPSAAVLDLSVPRVPHHPVHQGHHGFFSPTAAYMNALATR
ALPPTPPLMAAEHIKETAAEHLFKNVNWIKSVRAFTELPMPDQLLLLEESWKEFFILAMAQYLMPMNFAQLL
FVYESENANREIVTIVAREVHAFQAVPNRLCHLNIDSTEYECLRAISLFRKSPPAASSTEDLANSSILTGSG
SPNSSASAESRGLLESSKVAAMHNDARNALHNYISRTHPNQPLRFQTLLGVVTLMHKVSSFTIEELFFRKTI
GDITIVRLISDMYSQRKI
>AmDsf/1-437
MINYRDCDITAVESGDRLLDIPCLVCGDRSSGKHYGIYSCDGCSGFFKRSIHSNRRYICKVQGAMKGRCPID
KTHRNQCRACRLAKCFEANMNRDAVQHERGPRKPKQPQQQSPIAPPLHNDRLRAGLSSPYVLLHQRKFRCDQ
RFSPYPRPVALVQKPPEDSSSPAPLALPHSPSTTTLYSAPSSVALTPQPPLLQILMSAEQCQELVWNAPLQS
ETEYSLEQTEGSHNSSGTLPNPNPTRELLQETTARLLFMAVRWVCCLPLFQSLSKNDQLLLLEGSWTQLFLL
HLAQWSISWNITGLLEDEQVRARLPDEATTNQQLITIQDTICRFRQLSPDRSEWGCMKAVALFTPETEGLHA
TESIKMLQDQAQCILGDYTKSCYQRQPGRSGTLMHVVGRLTSIFPKLVERLFFHETIGEIPISRLLVDMYQM
KGHTN
>DmDsf/1-693
MGTAGDRLLDIPCKVCGDRSSGKHYGIYSCDGCSGFFKRSIHRNRIYTCKATGDLKGRCPVDKTHRNQCRAC
RLAKCFQSAMNKDAVQHERGPRKPKLHPQLHHHHHHAAAAAAAAHHAAAAHHHHHHHHHAHAAAAHHAAVAA
AAASGLHHHHHAMPVSLVTNVSASFNYTQHISTHPPAPAAPPSGFHLTASGAQQGPAPPAGHLHHGGAGHQH
ATAFHHPGHGHALPAPHGGVISNPGGNSSAISGSGPGSTLPFPSHLLHHNLIAEAASKLPGITATAVAAVVS
STSTPYASAAQASSPSSNNHNYSSPSPSNSIQSISSIGSRSGGGEEGLSLGSESPRVNVETETPSPSNSPPL
SAGSISPAPTLTTSSGSPQHRQMSRHSLSEATTPPSHASLMICASNNNNNNNNNNNNNNGEHKQSSYTSGSP
TPTTPTPPPPRSGVGSTCNTASSSSGFLELLLSPDKCQELIQYQVQHNTLLFPQQLLDSRLLSWEMLQETTA
RLLFMAVRWVKCLMPFQTLSKNDQHLLLQESWKELFLLNLAQWTIPLDLTPILESPLIRERVLQDEATQTEM
KTIQEILCRFRQITPDGSEVGCMKAIALFAPETAGLCDVQPVEMLQDQAQCILSDHVRLRYPRQATRFGRLL
LLLPSLRTIRAATIEALFFKETIGNVPIARLLRDMYTMEPAQVDK
>Human TLX/1-385
MSKPAGSTSRILDIPCKVCGDRSSGKHYGVYACDGCSGFFKRSIRRNRTYVCKSGNQGGCPVDKTHRNQCRA
CRLKKCLEVNMNKDAVQHERGPRTSTIRKQVALYFRGHKEENGAAAHFPSAALPAPAFFTAVTQLEPHGLEL
AAVSTTPERQTLVSLAQPTPKYPHEVNGTPMYLYEVATESVCESAARLLFMSIKWAKSVPAFSTLSLQDQLM
LLEDAWRELFVLGIAQWAIPVDANTLLAVSGMNGDNTDSQKLNKIISEIQALQEVVARFRQLRLDATEFACL
KCIVTFKAVPTHSGSELRSFRNAAAIAALQDEAQLTLNSYIHTRYPTQPCRFGKLLLLLPALRSISPSTIEE
VFFKKTIGNVPITRLLSDMYKSSDI
>AmPNR/1-394
MEDNSRGESLCKVCGDKASGKHYGVPSCDGCRGFFKRSIRRNLDYVCKENGRCIVDVSRRNQCQACRFTKCL
QVNMKRDAVQHERAPRNTSSLVAAARRGPSGLYPGIPHVYHAASNPYHPLLYPALFPFKPTMSAFTPSVAHF
LPRSPTTESLTVNAAKEDEVTSSEEAGSIDARIEPKEELASARLTPSSIANPASTEYISSFSILPTENIYEF
AAKLLFFAVRWARSIHSFLQLPYRDQTILLEESWSELFVLTAAQWNFPVEEAALVPNDLSSERKETLVDEAR
KLRELLAKCALLRVDHSEYACLKAIVLFKGESRGLCEPGRITALQEQTVAVFCERDARRVGRLLLLLPSARA
LCRSTLQELLFKPTVGDVSVERLLGDMVSALRPT
>AmHr78/1-548
MDRDHREQHDHDNINENEEEQKVGMDFRNLSRHHGVDHVADVEIERDMEVDQNDRIENLQDDKLDQNIGRNF
HNLAHHPAMRDMERDQNNHIDNMHDDKIDHKMGRDFRNLSSHTGIVHLHSGLEHLNNVDVEVKLARDVRGSL
GLGLSLELCVVCGDRASGRHYGAISCEGCKGFFKRSIRKQLGYQCRGSKSCEVTKHHRNRCQYCRLQKCLAM
GMRSDSVQHERKPVLGESAGAKVGNRSPRVKPEPQQSISETPPTWEQPESPSMEDQSSDSDLSDALTLARER
LLISHALDSMAKLIGESVNGSSEPEEEWTGQLISERHTLFELRAPSPAPAYLSIHYICESAARLLFLSVHWA
RGIPAFQALPSEVQTTLVRSSWGQLFTLGLAQCAYTLSLPSILTSIINHLQASIAQEKITASKVKSVTEHIC
RLQDCVSSLHKLQVDSIEYAYLKALTLFSADNVLAGVWRKKVEVLQEAAWTELQQRVGSNRLPRLLLRLAPL
RSINPRVLEDLFFAGLIGRVSVASVVPYILTMQDYKAEPESHMG
>Tenebrio Hr78/1-489
MDIEHSIKSEILSDKIRNNSNNLCLTVELCVVCGDRASGRHYGAISCEGCKGFFKRSIRKQLGYQCRGSKNC
EVTKHHRNRCQYCRLQKCLACGMRSDSVQHERKPIIDKKDYSNNIGNNYNSNSVNKIFIRKDLSTDSPGLLP
APFNPCDLGLQFLNKRIGNSGSSDLPYHLSPSQASIEDDVSMDSTNTGAGELSDALIMARDKQLISKALDTM
ARVQCLNGTDLSSLTAADEKCYEYEGPILQEQHISFNLQIPGPVPPYLNIHYICESGSRLLFLSIHWTRNIP
AFQYLTTETQITLLRGCWAELFTLGLAQCSQTLSLSTILSALISHLHTLIAQDKMSATKVKQVSDHIVKLQD
YANTMNRLNVDEHEYAYLKAITLFSADQPDILLRKHVEKLQEKSFQALKTYVHNSFPDDTDRFPRLLLRLPP
LRGLEPLVLEELFFAGLIGQVQIDSVIPYILRMGNGMPTPTSNRHVKSEQMEEFMCK
>Human TR2/1-603
MATIEEIAHQIIEQQMGEIVTEQQTGQKIQIVTALDHNTQGKQFILTNHDGSTPSKVILARQDSTPGKVFLT
TPDAAGVNQLFFTTPDLSAQHLQLLTDNSPDQGPNKVFDLCVVCGDKASGRHYGAVTCEGCKGFFKRSIRKN
LVYSCRGSKDCIINKHHRNRCQYCRLQRCIAFGMKQDSVQCERKPIEVSREKSSNCAASTEKIYIRKDLRSP
LTATPTFVTDSESTRSTGLLDSGMFMNIHPSGVKTESAVLMTSDKAESCQGDLSTLANVVTSLANLGKTKDL
SQNSNEMSMIESLSNDDTSLCEFQEMQTNGDVSRAFDTLAKALNPGESTACQSSVAGMEGSVHLITGDSSIN
YTEKEGPLLSDSHVAFRLTMPSPMPEYLNVHYIGESASRLLFLSMHWALSIPSFQALGQENSISLVKAYWNE
LFTLGLAQCWQVMNVATILATFVNCLHNSLQQDKMSTERRKLLMEHIFKLQEFCNSMVKLCIDGYEYAYLKA
IVLFSPDHPSLENMELIEKFQEKAYVEFQDYITKTYPDDTYRLSRLLLRLPALRLMNATITEELFFKGLIGN
IRIDSVIPHILKMEPADYNSQIIGHSI
>Human TR4/1-615
MTSPSPRIQIISTDSAVASPQRIQGSEPASGPLSVFTSLNKEKIVTDQQTGQKIQIVTAVDASGSPKQQFIL
TSPDGAGTGKVILASPETSSAKQLIFTTSDNLVPGRIQIVTDSASVERLLGKTDVQRPQVVEYCVVCGDKAS
GRHYGAVSCEGCKGFFKRSVRKNLTYSCRSSQDCIINKHHRNRCQFCRLKKCLEMGMKMESVQSERKPFDVQ
REKPSNCAASTEKIYIRKDLRSPLIATPTFVADKDGARQTGLLDPGMLVNIQQPLIREDGTVLLATDSKAET
SQGALGTLANVVTSLANLSESLNNGDTSEIQPEDQSASEITRAFDTLAKALNTTDSSSSPSLADGIDTSGGG
SIHVISRDQSTPIIEVEGPLLSDTHVTFKLTMPSPMPEYLNVHYICESASRLLFLSMHRARSIPAFQGLGQD
CNTSLVRACWNELFTLGLAQCAQVMSLSTILAAIVNHLQNSIQEDKLSGDRIKQVMEHIWKLQEFCNSMANW
DIDGYEYAYLKAIVLFSPDHPGLTSTSQIEKFQEKAQMELQDYVQKTYSEDTYRLARILVRLPALRLMSSNI
TEELFFTGLIGNVSIDSIIPYILKMETAEYNGQITGVSL
>Bombyx Hr78/1-461
MDGQDQLEMKFSSGSDVGGLELCIVCGDRASGRHYGAISCEGCKGFFKRSIRKKLGYQCRGTMNCEVTKHHR
NRCQYCRLQKCLACGMRSDFQHERKPIVDKNKSEPRDGLADRQAAYSKLLGLASQAPSAQQLTPKEEAGEAF
GAVSPAPAINFALAAAVAFNKNSVSPYLNPGSPGDMEGARRQQIMLQTQLAKNLFKMGQFGAINEYLQSAYG
AAPEPPPPADDARPDEMEVCSVLVPGGGALPLHAACESAARLLAATARALAALPAATALPFEIQVTLFKKSW
AELFVLGLCKLSHEMSLGTLLPSMAGHLHAVLRERASGAAAAHAPLDDHAPEISVWDYTDERIAEIISLLSR
LQQLVAAMEQLRVTDREYAQLRALCFFSPDGAPACAAARLEEAQARVSRALGGGGRAARLLLQLPALRAFPP
AFIEDVFFVGFLGDVCIDDAIPYLLNAER
>DmHr78/1-601
MDGVKVETFIKSEENRAMPLIGGGSASGGTPLPGGGVGMGAGASATLSVELCLVCGDRASGRHYGAISCEGC
KGFFKRSIRKQLGYQCRGAMNCEVTKHHRNRCQFCRLQKCLASGMRSDSVQHERKPIVDRKEGIIAAAGGSS
TSGGGNGSSTYLSGKSGYQQGRGKGHSVKAESAATPPVHSAPATAFNLNENIFPMGLNFAELTQTLMFATQQ
QQQQQQQHQQSGSYSPDIPKADPEDDEDDSMDNSSTLCLQLLANSASNNNSQHLNFNAGEAPTALPTTSTMG
LIQSSLDMRVIHKGLQILQPIQNQLERNGNLSVKPECDSEAEDSGTEDAVDAELEHMELDFECGGNRSGGSD
FAINEAVFEQDLLTDVQCAFHVQPPTLVHSYLNIHYVCETGSRIIFLTIHTLRKVPVFEQLEAHTQVKLLRG
VWPALMAIALAQCQGQLSVPTIIGQFIQSTRQLADIDKIEPLKISKMANLTRTLHDFVQELQSLDVTDMEFG
LLRLILLFNPTLLQQRKERSLRGYVRRVQLYALSSLRRQGGIGGGEERFNVLVARLLPLSSLDAEAMEELFF
ANLVGQMQMDALIPFILMTSNTSGL
>AmUSP/1-427
MMKKEKPMMSVTAIIQGTQAQHWSRGNTWLSLDNSNMSMSSVGPQSPLDMKPDTASLINPGNFSPSGPNSPG
SFTAGCHSNLLSTSPSGQNKAVAPYPPNHPLSGSKHLCSICGDRASGKHYGVYSCEGCKGFFKRTVRKDLSY
ACREEKSCIIDKRQRNRCQYCRYQKCLAMGMKREAVQEERQRTKERDQSEVESTSSLHSDMPIERILEAEKR
VECKMEQQGNYENAVSHICNATNKQLFQLVAWAKHIPHFTSLPLEDQVLLLRAGWNELLIASFSHRSIDVKD
GIVLATGITVHRNSAQQAGVGTIFDRVLSELVSKMREMKMDRTELGCLRSIILFNPEVRGLKSIQEVTLLRE
KIYGALEGYCRVAWPDDAGRFAKLLLRLPAIRSIGLKCLEYLFFFKMIGDVPIDDFLVEMLESRSDP
>Locusta USP/1-389
MEGSERGISLENNLSISSMGPQSPLDMKPDTASLISSGSFSPTGGPNSPGSFTIGHSSLLNNSSSNQAKGSS
SQYPPNHPLSGSKHLCSICGDRASGKHYGVYSCEGCKGFFKRTVRKDLSYACREDKNCIIDKRQRNRCQYCR
YQKCLAMGMKREAVQEERQRTKERDQNEVESTSSLHTDMPVERILEAEKRVECKAENQVEYELVEWAKHIPH
FTSLPLEDQVLLLRAGWNELLIAAFSHRSVDVKDGIVLATGLTVHRNSAHQAGVGTIFDRVLTELVAKMREM
KMDKTELGCLRSVILFNPEVRGLKSAQEVELLREKVYAALEEYTRTTHPDEPGRFAKLLLRLPSLRSIGLKC
LEHLFFFRLIGDVPIDTFLMEMLESPSDS
>Tenebrio USP/1-408
MTMESTDRALSLDQNLSMGSLGAPHSPLDMKPDASTLGQNSPVSFASGHGSLLSFSPQGPPSGGTPNKSCGS
LYPPNHPLSGSKHLCSICGDRASGKHYGVYSCEGCKGFFKRTVRKDLSYACREEKNCIIDKRQRNRCQYCRY
QKCLNMGMKREAVQEERQRTKDRDTSEVESTSNMQAEMPLDRIIEAEKRIECTPAGGSGGVGEQHDGVNNIC
QATNKQLFQLVQWAKLIPHFTSLPMSDQVLLLRAGWNELLIAAFSHRSIQAQDAIVLATGLTVNKTSAHAVG
VGNIYDRVLSELVNKMKEMKMDKTELGCLRAIILYNPTCRGIKSVQEVEMLREKIYGVLEEYTRTTHPNEPG
RFAKLLLRLPALRSIGLKCSEHLFFFKLIGDVPIDTFLMEMLESPADA
>Human RXRA/1-462
MDTKHFLPLDFSTQVNSSLTSPTGRGSMAAPSLHPSLGPGIGSPGQLHSPISTLSSPINGMGPPFSVISSPM
GPHSMSVPTTPTLGFSTGSPQLSSPMNPVSSSEDIKPPLGLNGVLKVPAHPSGNMASFTKHICAICGDRSSG
KHYGVYSCEGCKGFFKRTVRKDLTYTCRDNKDCLIDKRQRNRCQYCRYQKCLAMGMKREAVQEERQRGKDRN
ENEVESTSSANEDMPVERILEAELAVEPKTETYVEANMGLNPSSPNDPVTNICQAADKQLFTLVEWAKRIPH
FSELPLDDQVILLRAGWNELLIASFSHRSIAVKDGILLATGLHVHRNSAHSAGVGAIFDRVLTELVSKMRDM
QMDKTELGCLRAIVLFNPDSKGLSNPAEVEALREKVYASLEAYCKHKYPEQPGRFAKLLLRLPALRSIGLKC
LEHLFFFKLIGDTPIDTFLMEMLEAPHQMT
>Human RXRB/1-533
MSWAARPPFLPQRHAAGQCGPVGVRKEMHCGVASRWRRRRPWLDPAAAAAAAVAGGEQQTPEPEPGEAGRDG
MGDSGRDSRSPDSSSPNPLPQGVPPPSPPGPPLPPSTAPTLGGSGAPPPPPMPPPPLGSPFPVISSSMGSPG
LPPPAPPGFSGPVSSPQINSTVSLPGGGSGPPEDVKPPVLGVRGLHCPPPPGGPGAGKRLCAICGDRSSGKH
YGVYSCEGCKGFFKRTIRKDLTYSCRDNKDCTVDKRQRNRCQYCRYQKCLATGMKREAVQEERQRGKDKDGD
GEGAGGAPEEMPVDRILEAELAVEQKSDQGVEGPGGTGGSGSSPNDPVTNICQAADKQLFTLVEWAKRIPHF
SSLPLDDQVILLRAGWNELLIASFSHRSIDVRDGILLATGLHVHRNSAHSAGVGAIFDRVLTELVSKMRDMR
MDKTELGCLRAIILFNPDAKGLSNPSEVEVLREKVYASLETYCKQKYPEQQGRFAKLLLRLPALRSIGLKCL
EHLFFFKLIGDTPIDTFLMEMLEAPHQLA
>Human RXRG/1-463
MYGNYSHFMKFPAGYGGSPGHTGSTSMSPSAALSTGKPMDSHPSYTDTPVSAPRTLSAVGTPLNALGSPYRV
ITSAMGPPSGALAAPPGINLVAPPSSQLNVVNSVSSSEDIKPLPGLPGIGNMNYPSTSPGSLVKHICAICGD
RSSGKHYGVYSCEGCKGFFKRTIRKDLIYTCRDNKDCLIDKRQRNRCQYCRYQKCLVMGMKREAVQEERQRS
RERAESEAECATSGHEDMPVERILEAELAVEPKTESYGDMNMENSTNDPVTNICHAADKQLFTLVEWAKRIP
HFSDLTLEDQVILLRAGWNELLIASFSHRSVSVQDGILLATGLHVHRSSAHSAGVGSIFDRVLTELVSKMKD
MQMDKSELGCLRAIVLFNPDAKGLSNPSEVETLREKVYATLEAYTKQKYPEQPGRFAKLLLRLPALRSIGLK
CLEHLFFFKLIGDTPIDTFLMEMLETPLQIT
>Aedes USP/1-484
MLKKEKPMLSVAAIIQAQGRWDRTLPLAGLAGFDAALVGHMGPVSPQDMKPDLKPDISLLNGSVGPFSPGNN
CGPASPGAFNQQVAAALQQQQQNVNSLNSQQSGGGGGAGGGTPTTPTNMSQQYPPNHPLSGSKHLCSICGDR
ASGKHYGVYSCEGCKGFFKRTVRKDLSYACREDKNCTIDKRQRNRCQYCRYQKCLACGMKREAVQEERQRSS
KFSIKSEEINSTSSVRDVTIERIHEAEQLSEQKSGDNAIPYLRVGSNSMIPPEYKGAVSHLCQMVNKQIYQL
IDFARRVPHFINLPRDDQVMLLRCGWNEMLIAAVAWRSMEYIETERSSDGSRITVRQPQLMCLGPNFTLHRN
SAQQAGVDTLFDRILCELGIKMKRLDVTRAELGVLKAIILFNPDIRGLKCQKEIDGMREKIYACLDEHCKQQ
HPSEDGRFAQLLLRLPALRSISLKCLDHLNFIRLLSDKHLDSFIVEMLDMPI
>Chironomus USP/1-552
MLKKEKPMMTVAAIIEQAQNRWMDHPLVYNSRSLQFQGSYCIDSSLLGHMGPLSPPDLKPDISLLNCNNNNN
NTNNNNSNSSHNNLNHHNTSPLPVLGANTFSPIQSLNNNGPSSPLSSIGNGSGTIVTFNQIKLQSPSPSNAS
SSSTLSGPLTTTPPATNANNILGMGNGNCGNTANGKQSQYPPNHPLSGSKHLCSICGDRASGKHYGVYSCEG
CKGFFKRTVRKDLSYACREERNCVIDKKQRNRCQYCRYQKCLNCGMKREAVQEERQRGGKSQKGDDMSISST
QSLVNNGPGRDITVERLMEADQMSEARCGDKSIQYLRVAASNTMIPPEYRAPVSAICAMVNKQVFQHMDFCR
RLPHFTKLPLNDQMYLLKQSLNELLILNIAYMSIQYVEPDRRNADGSLERRQISQQMCLSRNYTLGRNMAVQ
AGVVQIFDRILSELSVKMKRLDLDATELCLLKSIVVFNPDVRTLDDRKSIDLLRSRIYASLDEYCRQKHPNE
DGRFAQLLLRLPALRSISLKCLDHLFYFQLIDDKNVENSVIEEFHKLN
>DmUSP/1-508
MDNCDQDASFRLSHIKEEVKPDISQLNDSNNSSFSPKAESPVPFMQAMSMVHVLPGSNSASSNNNSAGDAQM
AQAPNSAGGSAAAAVQQQYPPNHPLSGSKHLCSICGDRASGKHYGVYSCEGCKGFFKRTVRKDLTYACRENR
NCIIDKRQRNRCQYCRYQKCLTCGMKREAVQEERQRGARNAAGRLSASGGGSSGPGSVGGSSSQGGGGGGGV
SGGMGSGNGSDDFMTNSVSRDFSIERIIEAEQRAETQCGDRALTFLRVGPYSTVQPDYKGAVSALCQVVNKQ
LFQMVEYARMMPHFAQVPLDDQVILLKAAWIELLIANVAWCSIVSLDDGGAGGGGGGLGHDGSFERRSPGLQ
PQQLFLNQSFSYHRNSAIKAGVSAIFDRILSELSVKMKRLNLDRRELSCLKAIILYNPDIRGIKSRAEIEMC
REKVYACLDEHCRLEHPGDDGRFAQLLLRLPALRSISLKCQDHLFLFRITSDRPLEELFLEQLEAPPPPGLA
MKLE
>Lucilia USP/1-467
MDNGEQDAGFRLAPMSPQEIKPDISLLNENNTSSYSPKPGSPNPFAIGLQAINAVAAANANNQNQMLQTTPP
QQQQYPPNHPLSGSKHLCSICGDRASGKHYGVYSCEGCKGFFKRTVRKDLTYACREDRNCIIDKRQRNRCQY
CRYQKCLACGMKREAVQEERQRGTRAANARAAGAGGGGGGGGGVSNVVGAGGEDFKPSSSLRDLTIERIIEA
EQKAESLSGDNVLPFLRVGNNSMVQHDYKGAVSHLCQMVNKQLYQMVEYARRTPHFTHLQREDQILLLKAGW
NELLIANVAWCSIESLDAEYASPGTVHDGSFGRRSPVRQPQQLFLNQNFSYHRNSAIKANVVSIFDRILSEL
SIKMKRLNIDRSELSCLKAIILFNPDIRGLKCRADVEVCREKIYACLDEHCRTEHPGDDGRFAQLLLRLPAL
RSISLKCLDHLFFFRLIGERALEELIAEQLEAPIC
>Bombyx USP/1-462
MSSVAKKDKRTMSVTALINRAWPMTPSPQQQQQMVPSTQHSNFLHAMATPSTTPNVELDIQWLNIESGFMSP
MSPPEMKPDTAMLDGFRDDSTPPPPFKNYPPNHPLSGSKHLCSICGDRASGKHYGVYSCEGCKGFFKRTVRK
DLTYACREDKNCIIDKRQRNRCQYCRYQKCLACGMKREAVQEERQRAARRTEDAHPSSSVQELSIERLLELE
ALVADSAEELQILRVGPESGVPAKYRAPVSSLCQIGNKQIAALIVWARDIPHFGQLEIDDQILLIKGSWNEL
LLFAIAWRSMEFLNDERENVDSRNTAPPQLICLMPGMTLHRNSALQAGVGQIFDRVLSELSLKMRSLRMDQA
ECVALKAIILLNPDVKGLKNKQEVDVLREKMFLCLDEYCRRSRGGEEGRFAALLLRLPALRSISLKSFEHLY
LFHLVAEGSVSSYIRDALCNHAPPIDTNIM
>Manduca USP/1-461
MSSVAKKDKRTMSVTALINRAWPLTPAPHQQQSMPSSQPSNFLQPLATPSTTPSVELDIQWLNIEPGFMSPM
SPPEMKPDTAMLDGLRDDSTPPPAFKNYPPNHPLSGSKHLCSICGDRASGKHYGVYSCEGCKGFFKRTVRKD
LTYACREDRNCIIDKRQRNRCQYCRYQKCLACGMKREAVQEERQRAARGTEDAHPSSSVQELSIERLLEIES
LVADPPEEFQFLRVGPESGVPAKYRAPVSSLCQIGNKQIAALVVWARDIPHFGQLELEDQILLIKNSWNELL
LFAIAWRSMEYLTDERENVDSRSTAPPQLMCLMPGMTLHRNSALQAGVGQIFDRVLSELSLKMRTLRMDQAE
YVALKAIILLNPDVKGLKNKPEVVVLREKMFSCLDEYVRRSRCAEEGRFAALLLRLPALRSISLKCFEHLYF
FHLVADTSIASYIHDALRNHAPSIDTSIL
>Heliothis USP/1-466
MSVAKKDKPTMSVTALINWARPLPPGQQQQPMTPTSPGNMLQPMATPSNLPTVDCSLDIQWLNLEGGFMSPM
SPPEMKPDTAMLDGLRDDSTPPPAFKNYPPNHPLSGSKHLCSICGDRASGKHYGVYSCEGCKGFFKRTVRKD
LTYACREERNCIIDKRQRNRCQYCRYQKCLACGMKREAVQEERQRAARGTEDAHPSSSVQVQELSIERLLEM
ESLVADPSEEFQFLRVGPDSNVPPKFRAPVSSLCQIGNKQIAALVVWARDIPHFSQLEMEDQILLIKGSWNE
LLLFAIAWRSMEFLTEERDGVDGTGNRTTSPPQLMCLMPGMTLHRNSALQAGVGQIFDRVLSELSLKMRTLR
VDQAEYVALKAIILLNPDVKGLKNRQEVEVLREKMFLCLDEYCRRSRSSEEGRFAALLLRLPALRSISLKSF
EHLFFFHLVADTSIAGYIRDALRNHAPPIDTNMM
>Choristoneura USP/1-472
MSSVAKKDKPTMSVTALINWARPAPPGPPQPQSASPAPAAMLQQLPTQSMQSLNHIPTVDCSLDMQWLNLEP
GFMSPMSPPEMKPDTAMLDGLRDDATSPPNFKNYPPNHPLSGSKHLCSICGDRASGKHYGVYSCEGCKGFFK
RTVRKDLSYACREERNCIIDKRQRNRCQYCRYQKCLACGMKREAVQEERQRNARGAEDAHPSSSVQVSDELS
IERLTEMESLVADPSEEFQFLRVGPDSNVPPRYRAPVSSLCQIGNKQIAALVVWARDIPHFGQLELDDQVVL
IKASWNELLLFAIAWRSMEYLEDERENGDGTRSTTQPQLMCLMPGMTLHRNSAQQAGVGAIFDRVLSELSLK
MRTLRMDQAEYVALKAIVLLNPDVKGLKNRQEVDVLREKMFSCLDDYCRRSRSNEEGRFASLLLRLPALRSI
SLKSFEHLYFFHLVAEGSISGYIREALRNHAPPIDVNAMM
>CeFax-1/1-419
MSDEDEPLNFSTSKATEESKEGILGVRSIFNTPLLFPPPMFNAGVISPHIAAALAMSFNQQRMNASVSPPLD
HTTISVNSFPSMGSVKTDSPPTASSPTLCCAVCGDVSSGKHYGILACNGCSGFFKRSVRRRLIYRCQAGTGN
CVVDKAHRNQCQACRLKKCLNKGMNKDAVQNERQPRNTATIRPALDMDPQNFFREYAGAVSAIMGHSNMMKR
EDSPSSASDGKTEDEKKDSLQETTMSQLESVLQWAQQFRLFTVLTNSEKRQIILTQWPRLLCISLCEQAEDV
SFDDHLTSLMLKFRRLDVSPAEFNCLKAITIFMKRELSLWRAGWDNRASIITVYPAGERGARLVAAALLLAE
HSVMGFGNCVIPLALVFSTKSRYVIQRHAINSLPACVPGGTSAHPVLRCSMGSRREKVI
