# Supplementary material for: Nuclear receptors of the honey bee: annotation and expression in the adult brain
Source: Insect Mol Biol. 2006 Oct 1;15(5):583–95. doi: 10.1111/j.1365-2583.2006.00679.x (PMC1847479; doi:10.1111/j.1365-2583.2006.00679.x)
Supplement: S2 — CLUSTAL X (1.83) multiple sequence alignment. [file imb0015-0583-s2.rtf]

S2. Alignment
CLUSTAL X (1.83) multiple sequence alignment


AmHr83/1-299          CKVCGDRSYGKHYGVYCCDGCSCFFKRSVRRGALFTCIAGGACFVDKARRNWCPYCRLKK
DmHr83/1-278          CAVCGDQSSGKHYGVSCCDGCSCFFKRSVRRGSSYACIALGNCVVDKARRNWCPSCRFQR
Amtll/1-377           CKVCRDHSSGKHYGIFACDGCAGFFKRSIRRNRQYVCKAKGGCMVDKTHRNQCRACRLAK
Tctll/1-406           CKVCGDFSSGKHYNIFACDGCAGFFKRSIRRNRQYVCKAKGSCIIDKTHRNQCRACRLKK
Dmtll/1-452           CKVCRDHSSGKHYGIYACDGCAGFFKRSIRRSRQYVCKSQGLCVVDKTHRNQCRACRLRK
Dvtll/1-450           CKVCRDHSSGKHYGIYACDGCAGFFKRSIRRSRQYVCKSQGLCVVDKTHRNQCRACRLRK
Human TLX/1-385       CKVCGDRSSGKHYGVYACDGCSGFFKRSIRRNRTYVCKSGGGCPVDKTHRNQCRACRLKK
AmDsf/1-437           CLVCGDRSSGKHYGIYSCDGCSGFFKRSIHSNRRYICKVQGRCPIDKTHRNQCRACRLAK
DmDsf/1-693           CKVCGDRSSGKHYGIYSCDGCSGFFKRSIHRNRIYTCKATGRCPVDKTHRNQCRACRLAK
AmPNR-like/1-394      CKVCGDKASGKHYGVPSCDGCRGFFKRSIRRNLDYVCKENGRCIVDVSRRNQCQACRFTK
AmHr51/1-660          CVVCGDTSSGKHYGILACNGCSGFFKRSVRRKLIYRCQAGGRCVVDKAHRNQCQACRLKK
Human PNR/1-410       CRVCGDSSSGKHYGIYACNGCSGFFKRSVRRRLIYRCQVGGMCPVDKAHRNQCQACRLKK
DmHr51/1-532          CVVCGDTSSGKHYGILACNGCSGFFKRSVRRKLIYRCQAGGRCVVDKAHRNQCQACRLKK
AmUSP/1-427           CSICGDRASGKHYGVYSCEGCKGFFKRTVRKDLSYACREEKSCIIDKRQRNRCQYCRYQK
Locusta USP/1-389     CSICGDRASGKHYGVYSCEGCKGFFKRTVRKDLSYACREDKNCIIDKRQRNRCQYCRYQK
Tenebrio USP/1-408    CSICGDRASGKHYGVYSCEGCKGFFKRTVRKDLSYACREEKNCIIDKRQRNRCQYCRYQK
Human RXRA/1-462      CAICGDRSSGKHYGVYSCEGCKGFFKRTVRKDLTYTCRDNKDCLIDKRQRNRCQYCRYQK
Human RXRB/1-533      CAICGDRSSGKHYGVYSCEGCKGFFKRTIRKDLTYSCRDNKDCTVDKRQRNRCQYCRYQK
Human RXRG/1-463      CAICGDRSSGKHYGVYSCEGCKGFFKRTIRKDLIYTCRDNKDCLIDKRQRNRCQYCRYQK
Aedes USP/1-484       CSICGDRASGKHYGVYSCEGCKGFFKRTVRKDLSYACREDKNCTIDKRQRNRCQYCRYQK
Chilo USP/1-552       CSICGDRASGKHYGVYSCEGCKGFFKRTVRKDLSYACREERNCVIDKKQRNRCQYCRYQK
DmUSP/1-508           CSICGDRASGKHYGVYSCEGCKGFFKRTVRKDLTYACRENRNCIIDKRQRNRCQYCRYQK
Lucilia USP/1-467     CSICGDRASGKHYGVYSCEGCKGFFKRTVRKDLTYACREDRNCIIDKRQRNRCQYCRYQK
Bombyx USP/1-462      CSICGDRASGKHYGVYSCEGCKGFFKRTVRKDLTYACREDKNCIIDKRQRNRCQYCRYQK
Manduca USP/1-461     CSICGDRASGKHYGVYSCEGCKGFFKRTVRKDLTYACREDRNCIIDKRQRNRCQYCRYQK
Heliothis USP/1-466   CSICGDRASGKHYGVYSCEGCKGFFKRTVRKDLTYACREERNCIIDKRQRNRCQYCRYQK
Chironomus USP/1-410  CSICGDRASGKHYGVYSCEGCKGFFKRTVRKDLTYACREERNCIIDKRQRNRCQYCRYQK
ChoristoneurUSP/1-472 CSICGDRASGKHYGVYSCEGCKGFFKRTVRKDLSYACREERNCIIDKRQRNRCQYCRYQK
AmHr78/1-548          CVVCGDRASGRHYGAISCEGCKGFFKRSIRKQLGYQCRGSKSCEVTKHHRNRCQYCRLQK
Tenebrio Hr78/1-489   CVVCGDRASGRHYGAISCEGCKGFFKRSIRKQLGYQCRGSKNCEVTKHHRNRCQYCRLQK
Bombyx Hr78/1-461     CIVCGDRASGRHYGAISCEGCKGFFKRSIRKKLGYQCRGTMNCEVTKHHRNRCQYCRLQK
DmHr78/1-601          CLVCGDRASGRHYGAISCEGCKGFFKRSIRKQLGYQCRGAMNCEVTKHHRNRCQFCRLQK
Human TR2/1-603       CVVCGDKASGRHYGAVTCEGCKGFFKRSIRKNLVYSCRGSKDCIINKHHRNRCQYCRLQR
Human TR4/1-615       CVVCGDKASGRHYGAVSCEGCKGFFKRSVRKNLTYSCRSSQDCIINKHHRNRCQFCRLKK
CeFax-1/1-419         CAVCGDVSSGKHYGILACNGCSGFFKRSVRRRLIYRCQAGGNCVVDKAHRNQCQACRLKK
                      * :* * : *:**.   *:**  ****:::    : *     * :   :** *  **  :

AmHr83/1-299          CFTVGMNTAAVQEERGPLPPPILSTGDTIRYEIAARIFFATVLAARQHREFSMLDLEEQN
DmHr83/1-278          CLAVGMNAAAVQEERGPAAPSPTPHSQALHFQILAQILVTCLRQAKANEQFALLDRCQQD
AmTll/1-377           CIQAGMNKDAVQHERGPVYCNNMAMSKIPVCEQAARLLFLNVHWARDLAVGTNLVIEDQL
Tctll/1-406           CQNVGMNKDAVQHERGPLCNNYPPLPQVPPCESAAQLIFMNVQWVRSIPAFTCLPLSDQL
Dmtll/1-452           CFEVGMNKDAVQHERGPAAVLDLSVPRVPHKETAAEHLFKNVNWIKSVRAFTELPMPDQL
Dvtll/1-450           CFEVGMNKDAVQHERGPAAVLDLSVPRVPHKETAAEHLFKNVNWIKSVRAFTELPMPDQL
Human TLX/1-385       CLEVNMNKDAVQHERGPLVSLAQPTPKYPHCESAARLLFMSIKWAKSVPAFSTLSLQDQL
AmDsf/1-437           CFEANMNRDAVQHERGPLQILMSAEQCQELQETTARLLFMAVRWVCCLPLFQSLSKNDQL
DmDsf/1-693           CFQSAMNKDAVQHERGPHASLMICASNNNNQETTARLLFMAVRWVKCLMPFQTLSKNDQH
AmPNR-like/1-394      CLQVNMKRDAVQHERAPLTVNAAKEDEVTSYEFAAKLLFFAVRWARSIHSFLQLPYRDQT
AmHr51/1-660          CMQMGMNKDAVQNERQPTVAPTTNSTNPPTYETSARLLFMAVKWAKNLPSFASLPFRDQV
Human PNR/1-410       CLQAGMNQDAVQNERQPITAETCAKLEP--HETSARLLFMAVKWAKNLPVFSSLPFRDQV
DmHr51/1-532          CLQMGMNKDDDSIDVTNMSPN---------YETSARLLFMAVKWAKNLPSFARLSFRDQV
AmUSP/1-427           CLAMGMKREAVQEERQRLEAEKRVECKMEQCNATNKQLFQLVAWAKHIPHFTSLPLEDQV
Locusta USP/1-389     CLAMGMKREAVQEERQRLEAEKRVECKAEN----------LVEWAKHIPHFTSLPLEDQV
Tenebrio USP/1-408    CLNMGMKREAVQEERQRIEAEKRIECTPAGCQATNKQLFQLVQWAKLIPHFTSLPMSDQV
Human RXRA/1-462      CLAMGMKREAVQEERQRLEAELAVEPKTETCQAADKQLFTLVEWAKRIPHFSELPLDDQV
Human RXRB/1-533      CLATGMKREAVQEERQRLEAELAVEQKSDQCQAADKQLFTLVEWAKRIPHFSSLPLDDQV
Human RXRG/1-463      CLVMGMKREAVQEERQRLEAELAVEPKTESCHAADKQLFTLVEWAKRIPHFSDLTLEDQV
Aedes USP/1-484       CLACGMKREAVQEERQRHEAEQLSEQKSGDCQMVNKQIYQLIDFARRVPHFINLPRDDQV
Chilo USP/1-552       CLNCGMKREAVQEERQRMEADQMSEARCGDCAMVNKQVFQHMDFCRRLPHFTKLPLNDQM
DmUSP/1-508           CLTCGMKREAVQEERQRIEAEQRAETQCGDCQVVNKQLFQMVEYARMMPHFAQVPLDDQV
Lucilia USP/1-467     CLACGMKREAVQEERQRIEAEQKAESLSGDCQMVNKQLYQMVEYARRTPHFTHLQREDQI
Bombyx USP/1-462      CLACGMKREAVQEERQRLELEALVADSAEECQIGNKQIAALIVWARDIPHFGQLEIDDQI
Manduca USP/1-461     CLACGMKREAVQEERQRLEIESLVADPPEECQIGNKQIAALVVWARDIPHFGQLELEDQI
Heliothis USP/1-466   CLACGMKREAVQEERQRLEMESLVADPSEECQIGNKQIAALVVWARDIPHFSQLEMEDQI
Chironomus/1-410      CLACGMKREAVQEERQRLEMESLVADTSEECQIGNKQIAALVVWARDIPHFSQLEMDDQV
ChoristoneurUSP/1-472 CLACGMKREAVQEERQRTEMESLVADPSEECQIGNKQIAALVVWARDIPHFGQLELDDQV
AmHr78/1-548          CLAMGMRSDSVQHERK-LDSMAKLIG----CESAARLLFLSVHWARGIPAFQALPSEVQT
Tenebrio Hr78/1-489   CLACGMRSDSVQHERK-LDTMARVQCLNGTCESGSRLLFLSIHWTRNIPAFQYLTTETQI
Bombyx Hr78/1-461     CLACGMRSD-FQHERK-LFKMGQFGAINEYLFKKSWAELFVLG----------LCKLSHE
Drosophila Hr78/1-601 CLASGMRSDSVQHERK-LERNGNLSVKPECCETGSRIIFLTIHTLRKVPVFEQLEAHTQV
Human TR2/1-603       CIAFGMKQDSVQCERKPMQTNGDVSRAFDTGESASRLLFLSMHWALSIPSFQALGQENSI
Human TR4/1-615       CLEMGMKMESVQSERKPQSAS-EITRAFDTCESASRLLFLSMHRARSIPAFQGLGQDCNT
CeFax-1/1-419         CLNKGMNKDAVQNERQPMGHSNMMKREDSPQETTMSQLESVLQWAQQFRLFTVLTNSEKR
                      *    *.    . :                           :           :      

AmHr83/1-299          KILRRGWAAAFVLRAAIWPIDLTNTVNDAIFAARAVISSLQPDRIEFSVLETLILCRPAE
DmHr83/1-278          AIFQVVWSEIFVLRASHWSLDIS-CGDEQLKRLICEAHQLRADVLELNFMESLILCRKAI
AmTll/1-377           TLLESSWRELFLLAAAQILPTLDPVEVTRFRETLAGFHAMSLDQHEYACIRAIVLFKASN
Tctll/1-406           LLLEESWLDLFVLGAAQFLPLMDFKEVADFQETLKKISQFQLDAHEFACLRAIVLFKTKT
Dmtll/1-452           LLLEESWKEFFILAMAQYLMPMNFREVHAFQEVLNQLCHLNIDSTEYECLRAISLFRKRG
Dvtll/1-450           LLLEESWKEFFILAMAQYLMPMNFREVHAFQAVPNRLCHLNIDSTEYECLRAISLFRKRG
Human TLX/1-385       MLLEDAWRELFVLGIAQWAIPVDASEIQALQEVVARFRQLRLDATEFACLKCIVTFKARS
AmDsf/1-437           LLLEGSWTQLFLLHLAQWSISWNIQQLITIQDTICRFRQLSPDRSEWGCMKAVALFTPEG
DmDsf/1-693           LLLQESWKELFLLNLAQWTIPLDLTEMKTIQEILCRFRQITPDGSEVGCMKAIALFAPAG
AmPNR-like/1-394      ILLEESWSELFVLTAAQWNFPVEEDEARKLRELLAKCALLRVDHSEYACLKAIVLFKGRG
AmHr51/1-660          ILLEEAWSELFLLNAVQWCLPLE-ADVRHLHDTLQRYKAVMVDPAEFACMKAIVLFRPRG
Human PNR/1-410       ILLEEAWSELFLLGAIQWSLPLD-METRVLQETISRFRALAVDPTEFACMKALVLFKPRG
DmHr51/1-532          ILLEESWSELFLLNAIQWCIPLDPADVRTLHEIFCKYKAVLVDPAEFACLKAIVLFRPRG
AmUSP/1-427           LLLRAGWNELLIASFSHRSIDVKDIFDRVLSELVSKMREMKMDRTELGCLRSIILFNPRG
Locusta USP/1-389     LLLRAGWNELLIAAFSHRSVDVKDIFDRVLTELVAKMREMKMDKTELGCLRSVILFNPRG
Tenebrio USP/1-408    LLLRAGWNELLIAAFSHRSIQAQDIYDRVLSELVNKMKEMKMDKTELGCLRAIILYNPRG
Human RXRA/1-462      ILLRAGWNELLIASFSHRSIAVKDIFDRVLTELVSKMRDMQMDKTELGCLRAIVLFNPKG
Human RXRB/1-533      ILLRAGWNELLIASFSHRSIDVRDIFDRVLTELVSKMRDMRMDKTELGCLRAIILFNPKG
Human RXRG/1-463      ILLRAGWNELLIASFSHRSVSVQDIFDRVLTELVSKMKDMQMDKSELGCLRAIVLFNPKG
Aedes USP/1-484       MLLRCGWNEMLIAAVAWRSMEYIELFDRILCELGIKMKRLDVTRAELGVLKAIILFNPRG
Chilo USP/1-552       YLLKQSLNELLILNIAYMSIQYVEIFDRILSELSVKMKRLDLDATELCLLKSIVVFNPRT
DmUSP/1-508           ILLKAAWIELLIANVAWCSIVSLDIFDRILSELSVKMKRLNLDRRELSCLKAIILYNPRG
Lucialia USP/1-467    LLLKAGWNELLIANVAWCSIESLDIFDRILSELSIKMKRLNIDRSELSCLKAIILFNPRG
Bombyx USP/1-462      LLIKGSWNELLLFAIAWRSMEFLNIFDRVLSELSLKMRSLRMDQAECVALKAIILLNPKG
Manduca USP/1-461     LLIKNSWNELLLFAIAWRSMEYLTIFDRVLSELSLKMRTLRMDQAEYVALKAIILLNPKG
Heliothis USP/1-466   LLIKGSWNELLLFAIAWRSMEFLTIFDRVLSELSLKMRTLRVDQAEYVALKAIILLNPKG
Chironomus USP/1-410  LLIKGAWNELLLFAIAWRSMEFLNIFDRVLSELSLKMRHLRMDQAEYVALKAIILLNPKG
ChoristoneurUSP/1-472 VLIKASWNELLLFAIAWRSMEYLEIFDRVLSELSLKMRTLRMDQAEYVALKAIVLLNPKG
AmHr78/1-548          TLVRSSWGQLFTLGLAQCAYTLSLEHICRLQDCVSSLHKLQVDSIEYAYLKALTLFSAVL
Tenebrio Hr78/1-489   TLLRGCWAELFTLGLAQCSQTLSLDHIVKLQDYANTMNRLNVDEHEYAYLKAITLFSAQP
Bombys Hr78/1-461     MSLGTLLPSMAGHLHAVLRERASGSLLSRLQQLVAAMEQLRVTDREYAQLRALCFFSPAP
DmHr78/1-601          KLLRGVWPALMAIALAQCQGQLSVNLTRTLHDFVQELQSLDVTDMEFGLLRLILLFNPLQ
Human TR2/1-603       SLVKAYWNELFTLGLAQCWQVMNVEHIFKLQEFCNSMVKLCIDGYEYAYLKAIVLFSPPS
Human TR4/1-615       SLVRACWNELFTLGLAQCAQVMSLEHIWKLQEFCNSMANWDIDGYEYAYLKAIVLFSPPG
CeFax-1/1-419         QIILTQWPRLLCISLCEQAEDVS--FDDHLTSLMLKFRRLDVSPAEFNCLKAITIFMKAG
                        .                          :               *   :. :       

AmHr83/1-299          TMNGIRLTSRAMDTAVEILARHLAGKTESSVRVAKLMLILPILTASCPRKLANDLFAPII
DmHr83/1-278          NAEYAVILGSHSKAALISLARYTLQQSN-YLRFGQLLLGLRQLCLRRFDCALSCMFRSVV
AmTll/1-377           GSTSP-------NTGSRLRDAAAVARLRDGAQLALGQRLSGASFGALS------------
Tctll/1-406           TTESAKISVIQDDAQMRLNKHVTTTYPKQPLRFGKILLLVSSTFRTISRTIEDLFFKKVI
Dmtll/1-452           LLESGKVAAMHNDARSALHNYIQRTHPSQPMRFQTLLGVVQLMHKVSSFTIEELFFRKTI
Dvtll/1-450           LLESSKVAAMHNDARNALHNYISRTHPNQPLRFQTLLGVVTLMHKVSSFTIEELFFRKTI
Human TLX/1-385       FRNAAAIAALQDEAQLTLNSYIHTRYPTQPCRFGKLLLLLPALRSISPSTIEEVFFKKTI
AmDsf/1-437           LHATESIKMLQDQAQCILGDYTKSCYQRQPGRSGTLMHVVGRLTSIFPKLVERLFFHETI
DmDSF/1-693           LCDVQPVEMLQDQAQCILSDHVRLRYPRQATRFGRLLLLLPSLRTIRAATIEALFFKETI
AmPNR-like/1-394      LCEPGRITALQEQTVAVFCERDAR-------RVGRLLLLLPSARALCRSTLQELLFKPTV
AmHr51/1-660          LKDSSQIENLQDQAQVMLGQHARAQQPGSPARFGRLLLLLPLLRTVPASRVELIYFHRTI
Human PNR/1-410       LKDPEHVEALQDQSQVMLSQHSKAHHPSQPVRFGKLLLLLPSLRFITAERIELLFFRKTI
DmHr51/1-532          LKDPAQIENLQDQA-----HHTKTQFTAQIARFGRLLLMLPLLRMISSHKIESIYFQRTI
AmUSP/1-427           LKSIQEVTLLREKIYGALEGYCRVAWPDDAGRFAKLLLRLPAIRSIGLKCLEYLFFFKMI
Locusta USP/1-389     LKSAQEVELLREKVYAALEEYTRTTHPDEPGRFAKLLLRLPSLRSIGLKCLEHLFFFRLI
Tenebrio USP/1-408    IKSVQEVEMLREKIYGVLEEYTRTTHPNEPGRFAKLLLRLPALRSIGLKCSEHLFFFKLI
Human RXRA/1-462      LSNPAEVEALREKVYASLEAYCKHKYPEQPGRFAKLLLRLPALRSIGLKCLEHLFFFKLI
Human RXRB/1-533      LSNPSEVEVLREKVYASLETYCKQKYPEQQGRFAKLLLRLPALRSIGLKCLEHLFFFKLI
Human RXRG/1-463      LSNPSEVETLREKVYATLEAYTKQKYPEQPGRFAKLLLRLPALRSIGLKCLEHLFFFKLI
Aedes USP/1-484       LKCQKEIDGMREKIYACLDEHCKQQHPSEDGRFAQLLLRLPALRSISLKCLDHLNFIRLL
Chilo USP/1-552       LDDRKSIDLLRSRIYASLDEYCRQKHPNEDGRFAQLLLRLPALRSISLKCLDHLFYFQLI
DmUSP/1-508           IKSRAEIEMCREKVYACLDEHCRLEHPGDDGRFAQLLLRLPALRSISLKCQDHLFLFRIT
Lucilia USP/1-467     LKCRADVEVCREKIYACLDEHCRTEHPGDDGRFAQLLLRLPALRSISLKCLDHLFFFRLI
Bombyx USP/1-462      LKNKQEVDVLREKMFLCLDEYCRRSRGGEEGRFAALLLRLPALRSISLKSFEHLYLFHLV
Manduca USP/1-461     LKNKPEVVVLREKMFSCLDEYVRRSRCAEEGRFAALLLRLPALRSISLKCFEHLYFFHLV
Heliothis USP/1-466   LKNRQEVEVLREKMFLCLDEYCRRSRSSEEGRFAALLLRLPALRSISLKSFEHLFFFHLV
Chironomus USP/1-410  LGNRQEVEVLREKMYSCLDEYCRRVRVSEEGRFASLLLRLPALRSISLKSFEHLFFFHLV
ChoristoneurUSP/1-47  LKNRQEVDVLREKMFSCLDDYCRRSRSNEEGRFASLLLRLPALRSISLKSFEHLYFFHLV
AmHr78/1-548          AGVWRKKVEVLQEAAWTELQQRVG-----SNRLPRLLLRLAPLRSINPRVLEDLFFAGLI
Tenebrio Hr78/1-489   DILLRKHVEKLQEKSFQALKTYVHNSPDDTDRFPRLLLRLPPLRGLEPLVLEELFFAGLI
Bombyx Hr78/1-461     ACAAARLEEAQARVSR----------LGGGGRAARLLLQLPALRAFPPAFIEDVFFVGFL
DmHr78/1-601          QRKERSLRGYVRRVQLYALSSLRRQGGGGEERFNVLVARLLPLSSLDAEAMEELFFANLV
Human TR2/1-603       LENMELIEKFQEKAYVEFQDYITKTYPDDTYRLSRLLLRLPALRLMNATITEELFFKGLI
Human TR4/1-615       LTSTSQIEKFQEKAQMELQDYVQKTYSEDTYRLARILVRLPALRLMSSNITEELFFTGLI
CeFax-1/1-419         WDNRASIITVYPAGERGARLVAAALLEHSVMGFGNCVIPLALVFSTKSYVIQRHAINSLP
                                                                                  

AmHr83/1-299          GDIDLEKVIASVR---
DmHr83/1-278          RDILKTL---------
AmTll/1-377           --TNVVSAKLYKYAGY
Tctll/1-406           RDTPIVAIISNMYKNQ
Dmtll/1-452           GDITIVRLISDMYSQR
Dvtll/1-450           GDITIVRLISDMYSQR
Human TLX/1-385       GNVPITRLLSDMYKSS
AmDsf/1-437           GEIPISRLLVDMYQMK
DmDSF/1-693           GNVPIARLLRDMYTME
AmPNR-like/1-394      GDVSVERLLGDMVSAL
AmHr51/1-660          GNTPMEKVLCDMYKN-
Human PNR/1-410       GNTPMEKLLCDMFKN-
DmHr51/1-532          GNTPMEKVLCDMYKN-
AmUSP/1-427           GDVPIDDFLVEMLESR
Locusta USP/1-389     GDVPIDTFLMEMLESP
Tenebrio USP/1-408    GDVPIDTFLMEMLESP
Human RXRA/1-462      GDTPIDTFLMEMLEAP
Human RXRB/1-533      GDTPIDTFLMEMLEAP
Human RXRG/1-463      GDTPIDTFLMEMLETP
AedesUSP/1-484        SDKHLDSFIVEMLDMP
ChiloUSP/1-552        DDKNVENSVIEEFHKL
DmUSP/1-508           SDRPLEELFLEQLEAP
Lucilia USP/1-467     GERALEELIAEQLEAP
Bombyx USP/1-462      AEGSVSSYIRDALCNH
Manduca USP/1-461     ADTSIASYIHDALRNH
Heliothis USP/1-466   ADTSIAGYIRDALRNH
Chironomus USP/1-410  ADSSIAGYIRDLLRHH
ChoristoneurUSP/1-472 AEGSISGYIREALRNH
AmHr78/1-548          GRVSVASVVPYILTMQ
Tenebrio Hr78/1-489   GQVQIDSVIPYILRMG
Bombyx Hr78/1-461     GDVCIDDAIPYLLNAE
DmHr78/1-601          GQMQMDALIPFILMTS
Human TR2/1-603       GNIRIDSVIPHILKME
Human TR4/1-615       GNVSIDSIIPYILKME
CeFax-1/1-419         ACVPGGTSAHPVLRCS
                                      
